# A comprehensive dataset on cultivated and spontaneously growing vascular plants in urban gardens

**DOI:** 10.1016/j.dib.2019.103982

**Published:** 2019-05-23

**Authors:** David Frey, Marco Moretti

**Affiliations:** a Swiss Federal Research Institute WSL, Biodiversity and Conservation Biology, Zürcherstrasse 111, 8903 Birmensdorf, Switzerland; b Institute of Terrestrial Ecosystems, Department of Environmental Systems Science, ETH Zurich, Universitätstrasse 16, 8092 Zürich, Switzerland

**Keywords:** Allotment, BetterGardens, Home gardens, Lawn, Neophytes, Urban biodiversity, Vegetation relevés

## Abstract

This article summarizes the data of a survey of vascular plants in 85 urban gardens of the city of Zurich, Switzerland. Data was acquired by two sampling methods: (i) a floristic inventory of entire garden lots based on repeated garden visits, including all vegetation periods; and (ii) vegetation relevés on two plots of standardized size (10 m^2^) per garden during the summer. We identified a total of 1081 taxa and report the origin status, i.e., whether a taxon is considered native or alien to Switzerland. Furthermore, the origin of a plant or garden population was estimated for each taxon and garden: each taxon in each garden was classified as being either cultivated or spontaneously growing. For each garden, the number of all native, cultivated, and spontaneously growing plant species is given, along with additional information, including garden area, garden type and the landscape-scale proportion of impermeable surface within a 500-m radius. The dataset is related to the research note entitled “Research Note: Self-reported habitat heterogeneity predicts plant species richness in urban gardens” [1]. It is also linked to a comprehensive dataset on biotic and abiotic soil data and as well as to a dataset on soil-surface dwelling and flying arthropods [2–6].

**Table:** Specifications table

Subject area	*Ecology, Conservation Biology*
More specific subject area	*Urban ecology*
Type of data	*Tables, graphs*
How data was acquired	*Floristic inventories and vegetation relevés*
Data format	*Raw and aggregated*
Experimental factors	*A stratified sampling design with two types of gardens, and two crossed (independent) gradients: an urban intensity and a garden management intensity/habitat spatial heterogeneity gradient.*
Experimental features	*Gardens were chosen following a stratified sampling design, based on the urban habitat mapping key of the city of Zurich. The (independent) strata included i) garden type (domestic vs. allotment); ii) a garden spatial heterogeneity/management intensity gradient, ranging from extensively managed gardens with a high vertical vegetation structure and a high proportion of native plant species, to intensively managed gardens with a low vertical vegetation structure and a high proportion of alien plant species; and iii) an urban intensity gradient, which ranged from densely to less densely built-up areas of the city.*
Data source location	*City of Zurich, Switzerland; 47°22′N, 8°33′E*
Data accessibility	https://doi.org/10.17632/452pj39jm2.2
Related research article	*Young, C., Frey, D., Moretti, M., & Bauer, N. (2019). Research Note: Garden-owner reported habitat heterogeneity predicts plant species richness in urban gardens. Landscape and Urban Planning, 185, 222–227.*https://doi.org/10.1016/j.landurbplan.2019.01.013*.*[1]

**Table:** 

**Value of the Data** • The data is comprehensive as it describes all vascular plants growing on entirely sampled garden lots with a high taxonomic resolution, and plants growing on standardized sampling plots. • The data can contribute to comparative studies of community assembly rules of spontaneously versus human assembled urban plant communities. • The data can contribute to comparative studies of garden floras to understand mechanisms of plant introductions and invasions. • The data can be used to investigate the effects of garden plants on diversity patterns of species of other trophic levels (e.g. herbivors, pollinators), for which data exists from the same gardens • The data can be used to investigate above-below ground interactions, as biotic and abiotic soil data exists in the same gardens.

## 1 Data

**Fig. 1:**
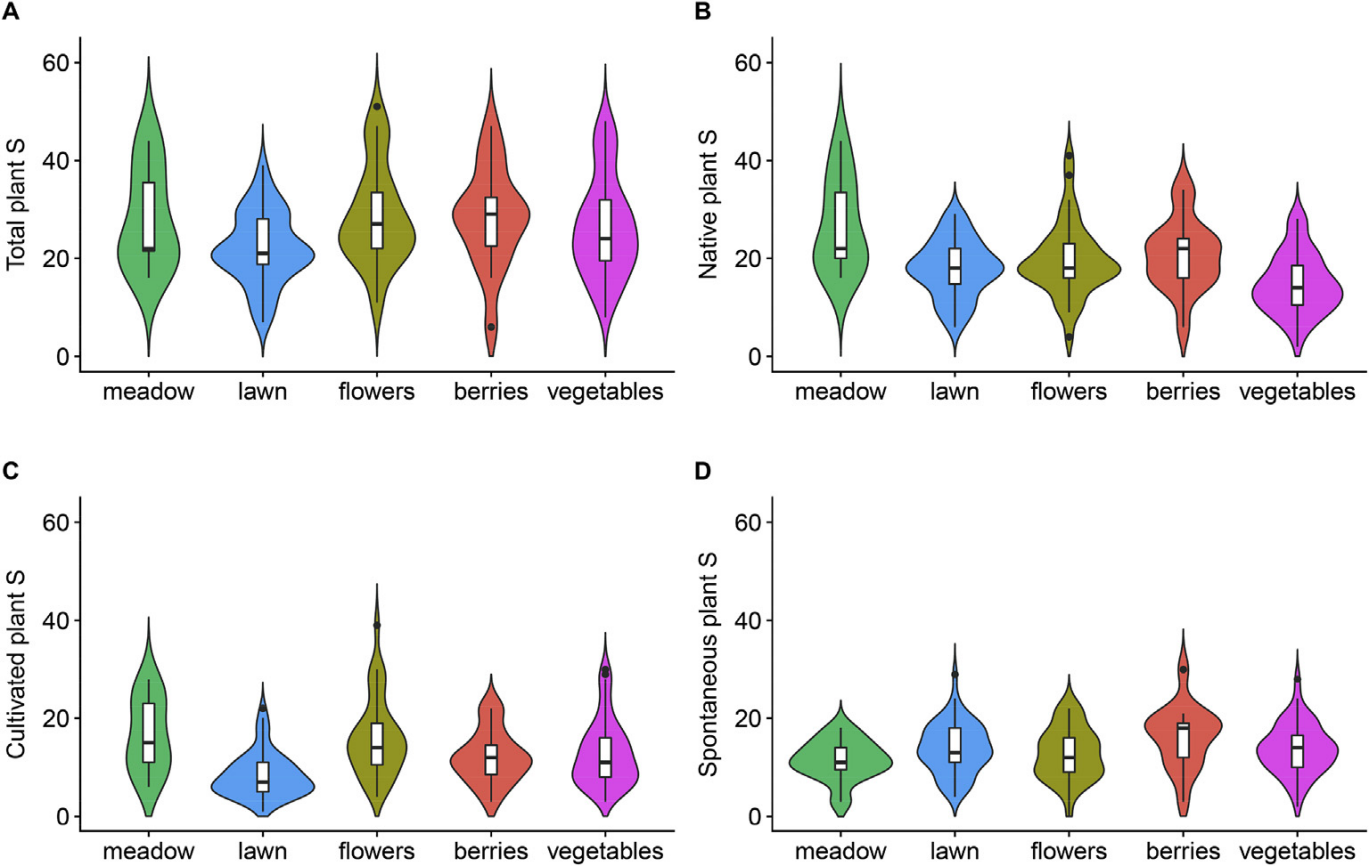
Violin plots illustrating the distribution of plant species richness of all (A), native (B), cultivated (C), and spontaneous (D) taxa growing on 10 m^2^ plots for each of the five land-use types based on all 85 gardens. Note the unequal sampling size among the land-use types: meadows (*N* = 11), lawns (*N* = 56), flower beds (*N* = 35), berry patches (*N* = 15) and vegetable beds (*N* = 47). Only the herbaceous vegetation layer was considered for meadows, lawn, and vegetables, and the tree layer was excluded altogether. Six plots were excluded due to pseudoreplication (e.g. two plots in lawn of the same garden).

**Fig. 2:**
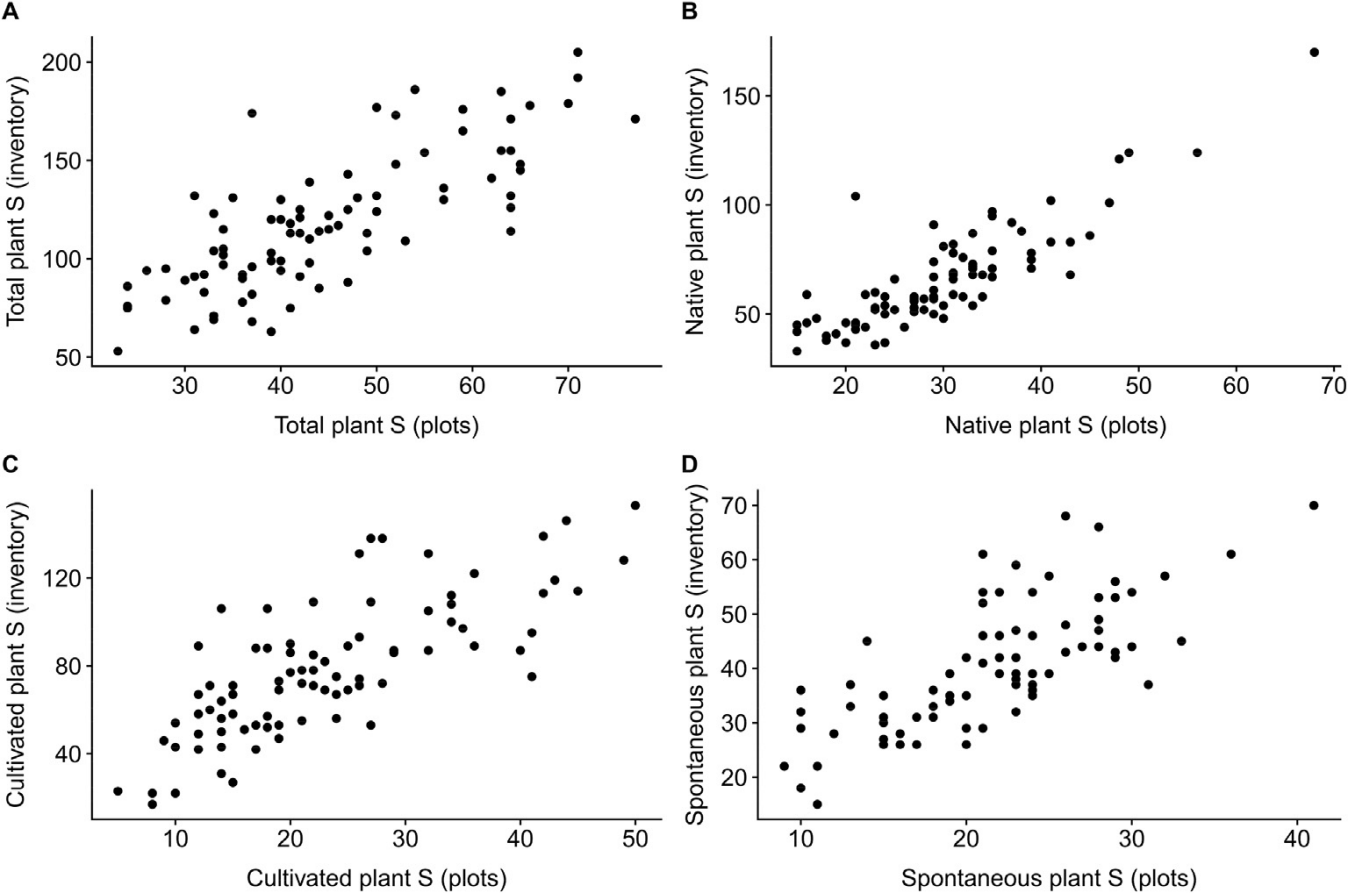
Comparison between the two sampling methods: Species richness (S) of plants growing in the two 10 m^2^ sampling plots versus plant species richness of the entire garden lot, based on data of all 85 urban gardens. Species richness of all (A), native (B), cultivated (C) and spontaneous (D) taxa are plotted. Note the different scales of the axes.

This article presents data of a survey of vascular plants in 85 urban gardens in the city of Zurich, Switzerland. Two garden types were investigated: allotment (*N* = 42) and domestic gardens (*N* = 43). In each garden, we applied two sampling methods: a floristic inventory of entire garden lots (mean area ±SD: 312 ± 155 m^2^) and sampling on plots of a standardized size (2 × 10 m^2^). The two plots were centred within the two main land-use types found in each garden: lawn, meadow, vegetable bed, flower bed or berry patch. We give the origin status for each of the 1081 taxa found, i.e., whether a taxon is considered native or alien to Switzerland (Appendix). Similarly, for each taxon and garden, we estimate the origin of each plant or garden population by classifying each taxon as either cultivated or spontaneously growing (Appendix). Species richness (i.e. number of taxa) of all (*S*_total_), native (*S*_native_), cultivated (*S*_cultivated_) and spontaneous (*S*_*s*pontaneous_) plants was computed for each garden and overall (Table 1). In addition, species richness levels of gardens and land-use types were visualized, and results of the two sampling methods were compared (Fig. 1, Fig. 2). For each garden, additional environmental data is given, such as garden type, area, and urbanization intensity, which was calculated as the landscape proportion of impervious (i.e. built and paved) surface within a 500-m radius (Table 1). The data is part of an inter- and transdisciplinary investigation of biodiversity, soil quality, ecosystem services and social value of urban gardens in Switzerland (www.bettergardens.ch). The data can be linked to biotic and abiotic soil data [2], [3], [4], to data of soil surface dwelling and flying arthropods, which were sampled in the same gardens and during the same period, and to arthropod and bird predation data [5], [6]. The raw data are available from Mendeley Data https://doi.org/10.17632/452pj39jm2.2 [7].

**Table 1:** Species richness (i.e. number of taxa) of all (*S*_total_), native (*S*_native_), cultivated (*S*_cultivated_) and spontaneous (*S*_*s*pontaneous_) vascular plants in 85 urban gardens in the City of Zurich, Switzerland. The data are based on a complete floristic inventory of garden lots. For each garden, garden type, area and landscape proportion of sealed surface within a 500-m radius is given. Sealed surface was defined as built and paved land-cover. Note that plants can belong to more than one category (i.e., native, cultivated, spontaneous), since a plant can occur spontaneously in one garden while it is cultivated in another, and both cultivated and spontaneous plants can be native.

Garden Id	Garden type	Garden area (m2)	Landscape proportion of sealed surface (500-m scale)	*S*_total_	*S*_native_	*S*_cultivated_	*S*_spontaneous_
1	allotment	210.3	0.190	171	103	114	57
2	home	285.1	0.393	148	78	109	39
3	allotment	183.9	0.630	115	52	78	37
4	home	399.5	0.233	145	81	89	56
5	home	666.8	0.272	165	87	108	57
6	allotment	169.5	0.390	126	68	87	39
7	home	238.1	0.671	69	53	22	47
8	home	473.5	0.428	79	40	51	28
9	allotment	198.8	0.200	97	61	43	54
10	allotment	246.1	0.536	179	99	113	66
11	home	659.2	0.434	89	50	54	35
12	home	476.2	0.429	130	65	88	42
13	allotment	197.0	0.432	91	56	53	38
14	allotment	172.6	0.218	113	61	69	44
15	allotment	256.1	0.285	78	43	47	31
16	allotment	307.5	0.243	120	59	86	34
17	home	346.9	0.210	71	37	42	29
18	allotment	179.6	0.210	109	60	67	42
19	home	510.4	0.812	114	69	75	39
20	allotment	496.0	0.208	139	91	78	61
21	allotment	223.9	0.273	155	84	128	27
22	home	170.2	0.726	64	52	17	47
23	allotment	212.5	0.207	113	55	72	41
24	allotment	205.3	0.290	148	83	87	61
25	home	695.7	0.295	132	75	85	47
26	home	400.1	0.559	120	71	67	53
27	home	135.9	0.811	117	70	75	42
28	home	407.5	0.613	110	60	75	35
29	allotment	734.5	0.362	114	77	58	56
30	home	273.6	0.716	185	124	145	40
31	allotment	167.9	0.211	104	51	69	35
32	home	229.7	0.284	95	43	73	22
33	allotment	198.7	0.248	115	58	69	46
34	allotment	173.1	0.539	63	38	27	36
35	home	249.9	0.724	92	47	56	36
36	home	255.9	0.373	96	52	64	32
37	home	291.0	0.582	132	82	95	37
38	allotment	501.2	0.159	99	57	53	46
39	home	791.5	0.191	91	73	23	68
40	home	294.2	0.534	105	58	71	34
41	home	444.2	0.430	131	68	89	42
42	home	486.1	0.248	205	169	153	52
43	home	107.4	0.616	53	39	22	31
44	home	497.2	0.517	118	62	77	41
45	home	355.5	0.574	178	95	119	59
46	allotment	212.5	0.195	113	46	82	31
47	home	286.8	0.472	186	97	138	48
48	home	422.2	0.721	102	54	67	35
49	allotment	169.1	0.320	125	53	88	37
50	home	150.9	0.613	92	37	74	18
51	allotment	184.5	0.360	122	49	100	22
52	home	366.7	0.497	174	107	138	36
53	allotment	410.3	0.818	125	80	71	54
54	allotment	179.6	0.187	75	52	31	44
55	allotment	240.4	0.111	124	70	71	53
56	home	438.9	0.249	171	123	122	49
57	home	121.7	0.814	98	50	72	26
58	home	551.4	0.383	143	102	89	54
59	home	455.3	0.441	75	45	43	32
60	allotment	220.2	0.129	99	60	57	42
61	allotment	150.4	0.109	88	48	51	37
62	home	291.7	0.663	103	55	60	43
63	allotment	370.9	0.834	130	67	86	44
64	allotment	201.6	0.548	155	75	112	43
65	home	479.6	0.215	121	68	93	28
66	allotment	232.4	0.423	123	50	90	33
67	allotment	507.4	0.279	176	87	106	70
68	allotment	494.2	0.461	94	61	49	45
69	allotment	200.7	0.287	132	50	106	26
70	allotment	242.4	0.183	192	116	139	53
71	home	287.2	0.215	177	84	131	46
72	allotment	486.1	0.353	104	62	58	46
73	home	277.1	0.697	76	46	46	30
74	allotment	150.9	0.308	83	42	50	33
75	allotment	177.8	0.399	154	70	109	45
76	allotment	180.5	0.491	131	47	105	26
77	allotment	231.5	0.405	86	32	71	15
78	home	244.8	0.651	173	91	131	42
79	home	207.9	0.621	94	57	52	42
80	allotment	186.4	0.252	141	71	87	54
81	allotment	110.2	0.439	68	38	42	26
82	allotment	177.8	0.421	136	69	97	39
83	home	288.0	0.369	90	70	55	35
84	home	281.6	0.256	85	46	56	29
85	home	438.7	0.216	82	68	53	29
Mean ± SD		311.6 ±155.1	0.411 ±0.195	118.8 ±34.7	66.5 ±23.2	77.9 ±30.8	40.9 ±11.4
Range		107.4–791.5	0.109–0.834	53-205	32-169	17-153	15-70

## 2 Experimental design, materials and methods

### 2.1 Data source

The data was acquired in the city of Zurich, Switzerland (47°22′N, 8°33′E). Zurich is located in the temperate climate zone of Europe, with a mean annual temperature of 9.3 °C (1981–2010) and mean annual precipitation of 1134 mm [8]. It harbours a population of 0.4 million in an area of approximately 92 km^2^, placing it in the globally most common city class [9].

### 2.2 Garden selection

We collected floristic data in 85 urban gardens (43 domestic and 42 allotment gardens). We defined a domestic garden to be a garden directly adjacent to a single-occupancy or terraced house. Our definition of allotment gardens encompasses spatially clustered garden lots on public land, managed by associations and leased to leisure gardeners as lots of 100m^2^–200m^2^ [10].

Gardens were chosen following a stratified sampling design, based on visual criteria defined by the urban habitat mapping key to the city of Zurich [11]. Potentially suitable gardens were identified based on the habitat map of the city of Zurich, aerial images and during field visits. We approached the garden owners initially by letter and thereafter by phone to arrange a visit. If no phone number was available, owners were approached personally. The (independent) strata included i) garden type (domestic vs. allotment), ii) a garden spatial heterogeneity/management intensity gradient, ranging from extensively managed gardens with a high vertical vegetation structure and a high proportion of native plant species, to intensively managed gardens with a low vertical vegetation structure and a high proportion of alien plant species [11], and iii) an urban intensity gradient, which ranged from densely to less densely built-up areas of the city. The urban intensity gradient was quantified as the proportional area of impervious (i.e. built and paved) surface within a 500-m radius around each garden lot (Table 1).

Variance in garden area was kept small and no novel gardens were included. To assure statistical independence among observations, no adjacent garden lots were sampled, and gardens were distributed across the city to include all urban districts. Additionally, with two exceptions, only one garden lot was sampled per allotment garden area. The average pairwise distance between gardens was 4.5 km (SD ± 2.2 km, min.-max. 0.1–11 km).

### 2.3 Floristic data of garden lots

A complete floristic inventory of each garden lot was made during repeated garden visits in 2015, based on the standard determination literature of the Swiss, resp. European (garden) flora [12], [13], [14], [15], [16], [17]. Potted plants were included in the inventory. Abundance of each taxon was estimated semi-quantitatively on a six-point scale: 1 (1–10 individuals), 2 (11–25 ind.), 3 (26–50 ind.), 4 (51–100 ind.), 5 (101–250 ind.) and 6 (>250 ind.). To account for the different vegetation periods, gardens were visited three times in March/April, May, and July/August. To standardize the sampling effort among gardens, the duration of each visit was restricted to about 1.5 h. Note that the early spring flowering genus *Crocus* L. was missed. The species richness of each garden is given in Table 1. The list of all taxa and the number of observations per taxon is given in the Appendix. The raw data is available from Mendeley Data https://doi.org/10.17632/452pj39jm2.2 [7].

### 2.4 Sampling on standardized plots (vegetation relevés)

In each garden, in addition to the floristic inventory, plant species were sampled on two circular plots of 10 m^2^ each during the summer. The methodology was based on the survey of angiosperm diversity of the Swiss Biodiversity Monitoring program [18]. Potted plants were not included. On each plot, three vegetation layers (ground vegetation, shrub and tree layer) were roughly distinguished and within each layer, the percentage cover of each taxon was scored on an ordinal scale: 1 (<1% cover), 2 (1–5% cover), 3 (>5–25% cover), 4 (>25–50% cover), 5 (>50–75% cover), 6 (>75–100% cover). Note that due to the heterogenous vegetation structure of gardens, cover sums over 100% within a vegetation layer were allowed. The two plots were centred within the two main garden land-use types found in each garden: lawn, meadow, vegetable bed, flower bed or berry patch. In addition, the levels of soil disturbance were contrasted between the two selected land-use types [2]: in each garden, one of the plots had to be in a low soil disturbance land-use type with mostly perennial vegetation (e.g. lawn), while the other had to be in a high soil disturbance land-use type with mostly annual vegetation (e.g. a vegetable bed). The distribution of species richness in each land use type is given in Fig. 1. A comparison between the two sampling methods is given in Fig. 2. The plot-based data can be linked to urban soil data of land-use types [3]. The raw data is available from Mendeley Data https://doi.org/10.17632/452pj39jm2.2 [7].

### 2.5 Taxonomic treatment, origin status and origin of garden populations

Taxonomy largely followed the Checklist of the National Data and Information Centre of the Swiss Flora [19]. In addition, for cultivated ornamental plants, Huxley & Royal Horticultural Society [13], Jäger [14], and Cullen [15] were consulted. Taxa below the species level, taxa within species complexes, and cultivars were not consequently determined at the lowest possible taxonomic rank. They were mostly grouped into aggregates (e.g. *Taraxacum officinale* aggr.), Cultivar Groups (e.g. *Begonia* Semperflorens Cultorum Group), or labeleld CV as cultivars without further distinction (e.g. *Rosa* CV).

Juillerat et al. [19] were followed to assess the origin status of species, i.e. whether a taxon is considered native or alien to Switzerland. Our definition of native plants encompasses archeophytes, which are taxa introduced to Switzerland before 1500, and neophytes of European origin that have colonized Switzerland spontaneously. A more detailed description of the origin status is given in the raw data [7]. Cultivar groups not derived from native plants were considered to be alien.

The origin of a plant individual or “population” in a garden, i.e. whether a plant or a group of plants was cultivated, or whether it occurred spontaneously, was determined by consulting the Flora of the City of Zurich [20] and/or by asking the garden owners. Intentionally introduced plants, which subsequently formed self-sustaining local populations, were considered to be cultivated. Plants that spontaneously colonized a garden and were subsequently tolerated or even locally favored by the garden owner/tenant were considered to be spontaneous plants. The origin of rare native plants was always verified by asking the garden owners. Meadow and lawn plants originating from seeding were considered to be cultivated plants. In lawns and meadows not deliberately enriched with herbs, only grasses employed in landscaping were considered to be cultivated [13].

It is important to note that the origin of a garden population can not always be unambiguously retraced and should therefore be interpreted with caution: especially in the case of lawn and grassland plants. Note that a taxon can belong to more than one category (native, cultivated, spontaneous), since a species may occur spontaneously in one garden, while it is cultivated in another, and both cultivated and spontaneous plants can be native.

## Figures and Tables

**Table:** List and number of observations of vascular plants taxa in 85 urban gardens in the City of Zurich, Switzerland, based on the floristic inventory. OS, origin status (A, alien/ N, native); *N*_cult_, number of observations of garden populations of anthropic origin; *N*_spont_, number of observations of spontaneously occurring garden populations; *N*_total_, total number of observations of a taxon. Note that the early spring flowering genus *Crocus* L. was missed.

Taxon name	Authority	Family	OS	*N*_cult._	*N*_spont._	*N*_total_
Abelmoschus esculentus	(L.) Moench	Malvaceae	A	1	0	1
Abies alba	Mill.	Pinaceae	N	2	0	2
Abutilon × hybridum	Voss	Malvaceae	A	2	0	2
Acanthus mollis	L.	Acanthaceae	A	7	0	7
Acer × conspicuum	van Gelderen & Oterdoom	Sapindaceae	A	1	0	1
Acer campestre	L.	Sapindaceae	N	3	14	17
Acer palmatum	Thunb.	Sapindaceae	A	19	0	19
Acer platanoides	L.	Sapindaceae	N	1	6	7
Acer pseudoplatanus	L.	Sapindaceae	N	3	13	16
Achillea filipendulina	Lam.	Asteraceae	A	5	0	5
Achillea millefolium aggr.		Asteraceae	N	14	2	16
Achillea ptarmica	L.	Asteraceae	N	3	0	3
Acinos arvensis	(Lam.) Dandy	Lamiaceae	N	1	0	1
Aconitum napellus	L.	Ranunculaceae	N	8	0	8
Actinidia arguta	(Siebold & Zucc.) Planch. ex Miq.	Actinidiaceae	A	5	0	5
Actinidia chinensis	Planch.	Actinidiaceae	A	8	0	8
Actinidia kolomikta	(Rupr. & Maxim.) Maxim.	Actinidiaceae	A	1	0	1
Adenophora liliifolia	(L.) A. DC.	Campanulaceae	N	1	0	1
Adiantum capillus-veneris	L.	Pteridaceae	N	1	0	1
Aegopodium podagraria	L.	Apiaceae	N	0	26	26
Aeonium haworthii	Webb & Berthel.	Crassulaceae	A	1	0	1
Aesculus hippocastanum	L.	Sapindaceae	A	3	0	3
Aethusa cynapium	L.	Apiaceae	N	0	2	2
Agapanthus praecox	Willd.	Amaryllidaceae	A	3	0	3
Agastache mexicana	(Kunth) Lint & Epling	Lamiaceae	A	3	0	3
Agave americana	L.	Asparagaceae	A	1	0	1
Ageratum houstonianum	Mill.	Asteraceae	A	1	0	1
Agrimonia eupatoria	L.	Rosaceae	N	0	4	4
Agrostis capillaris	L.	Poaceae	N	3	0	3
Agrostis gigantea	Roth	Poaceae	N	0	2	2
Agrostis stolonifera	L.	Poaceae	N	38	0	38
Ailanthus altissima	(Mill.) Swingle	Simaroubaceae	A	0	2	2
Ajuga reptans	L.	Lamiaceae	N	8	34	42
Akebia quinata	(Houtt.) Decne.	Lardizabalaceae	A	1	0	1
Alcea rosea	L.	Malvaceae	A	35	0	35
Alchemilla mollis	(Buser) Rothm.	Rosaceae	A	31	0	31
Alchemilla vulgaris aggr.		Rosaceae	N	2	1	3
Alisma plantago-aquatica	L.	Alismataceae	N	3	0	3
Alliaria petiolata	(M. Bieb.) Cavara & Grande	Brassicaceae	N	0	21	21
Allium carinatum	L.	Amaryllidaceae	N	1	0	1
Allium cepa	L.	Amaryllidaceae	A	20	0	20
Allium cristophii	Trautv.	Amaryllidaceae	A	18	0	18
Allium fistulosum	L.	Amaryllidaceae	A	2	0	2
Allium neapolitanum	Cirillo	Amaryllidaceae	A	1	0	1
Allium nigrum	L.	Amaryllidaceae	A	2	0	2
Allium porrum	L.	Amaryllidaceae	A	17	0	17
Allium schoenoprasum	L.	Amaryllidaceae	N	41	0	41
Allium siculum	Ucria	Amaryllidaceae	A	1	0	1
Allium tuberosum	Rottler ex Spreng.	Amaryllidaceae	A	1	0	1
Allium ursinum	L.	Amaryllidaceae	N	0	31	31
Allium vineale	L.	Amaryllidaceae	N	1	0	1
Aloe vera	(L.) Burm. f.	Asphodelaceae	A	1	0	1
Alopecurus pratensis	L.	Poaceae	N	2	0	2
Aloysia citriodora	Palau	Verbenaceae	A	7	0	7
Althaea officinalis	L.	Malvaceae	A	5	0	5
Amaranthus blitum aggr.		Amaranthaceae	N	0	20	20
Amaranthus caudatus	L.	Amaranthaceae	A	8	0	8
Amaranthus hybridus aggr.		Amaranthaceae	A	0	3	3
Amaranthus retroflexus	L.	Amaranthaceae	A	0	2	2
Amelanchier alnifolia	(Nutt.) Nutt. ex M. Roem.	Rosaceae	A	2	0	2
Amelanchier lamarckii	F. G. Schroed.	Rosaceae	A	11	0	11
Amelanchier ovalis	Medik.	Rosaceae	N	2	0	2
Anagallis arvensis	L.	Primulaceae	N	0	23	23
Anchusa officinalis	L.	Boraginaceae	N	1	0	1
Androsace septentrionalis	L.	Primulaceae	N	1	0	1
Androsace studiosorum	(Duby) Govaerts	Primulaceae	A	1	0	1
Anemone blanda	Schott & Kotschy	Ranunculaceae	A	7	0	7
Anemone coronaria	L.	Ranunculaceae	A	1	0	1
Anemone hupehensis	(Lemoine) Lemoine	Ranunculaceae	A	29	0	29
Anemone nemorosa	L.	Ranunculaceae	N	0	32	32
Anemone sylvestris	L.	Ranunculaceae	N	1	0	1
Anethum graveolens	L.	Apiaceae	A	8	0	8
Angelica archangelica	L.	Apiaceae	A	1	0	1
Angelica sylvestris	L.	Apiaceae	N	2	0	2
Anthemis tinctoria	(L.) J. Gay	Asteraceae	N	5	0	5
Anthericum ramosum	L.	Asparagaceae	N	2	0	2
Anthoxanthum odoratum	L.	Poaceae	N	6	0	6
Anthyllis vulneraria	L.	Fabaceae	N	1	0	1
Antirrhinum majus	L.	Plantaginaceae	A	27	0	27
Apium graveolens	L.	Apiaceae	A	22	0	22
Aquilegia skinneri	Hook.	Ranunculaceae	A	1	0	1
Aquilegia vulgaris	L.	Ranunculaceae	N	63	0	63
Arabidopsis thaliana	(L.) Heynh.	Brassicaceae	N	0	4	4
Arabis alpina subsp. caucasica	(Willd.) Briq.	Brassicaceae	A	2	0	2
Araucaria araucana	(Molina) K. Koch	Araucariaceae	A	1	0	1
Arctium lappa	L.	Asteraceae	N	1	3	4
Argyranthemum frutescens	(L.) Sch. Bip.	Asteraceae	A	3	0	3
Aristolochia clematitis	L.	Aristolochiaceae	N	1	0	1
Aristolochia macrophylla	Lam.	Aristolochiaceae	A	1	0	1
Aristolochia rotunda	L.	Aristolochiaceae	N	1	0	1
Armeria maritima	(Mill.) Willd.	Plumbaginaceae	A	4	0	4
Armoracia rusticana	G. Gaertn. & al.	Brassicaceae	N	16	0	16
Aronia melanocarpa	(Michx.) Elliott	Rosaceae	A	4	0	4
Arrhenatherum elatius	(L.) J. Presl & C. Presl	Poaceae	N	10	13	23
Artemisia abrotanum	L.	Asteraceae	A	6	0	6
Artemisia absinthium	L.	Asteraceae	N	6	0	6
Artemisia campestris	L.	Asteraceae	N	1	0	1
Artemisia dracunculus	L.	Asteraceae	A	1	0	1
Artemisia tridentata	Nutt.	Asteraceae	A	1	0	1
Artemisia vulgaris	L.	Asteraceae	N	6	0	6
Arum italicum	Mill.	Araceae	N	6	0	6
Arum maculatum	L.	Araceae	N	0	8	8
Aruncus dioicus	(Walter) Fernald	Rosaceae	N	3	4	7
Asarum europaeum	L.	Aristolochiaceae	N	1	0	1
Asclepias syriaca	L.	Apocynaceae	A	3	0	3
Asimina triloba	(L.) Dunal	Annonaceae	A	1	0	1
Asparagus densiflorus	(Kunth) Jessop	Asparagaceae	A	2	0	2
Asparagus officinalis	L.	Asparagaceae	N	8	0	8
Asphodeline lutea	(L.) Rchb.	Asphodelaceae	A	2	0	2
Asplenium ruta-muraria	L.	Aspleniaceae	N	0	2	2
Asplenium trichomanes	L.	Aspleniaceae	N	2	2	4
Aster amellus	L.	Asteraceae	N	1	0	1
Aster dumosus	Hoffm.	Asteraceae	A	2	0	2
Aster novae-angliae	L.	Asteraceae	A	15	0	15
Aster novi-belgii aggr.		Asteraceae	A	25	0	25
Astilbe Arendsii Group		Saxifragaceae	A	17	0	17
Astilboides tabularis	(Hemsl.) Engl.	Saxifragaceae	A	1	0	1
Astrantia major	L.	Apiaceae	N	2	0	2
Athyrium filix-femina	(L.) Roth	Athyriaceae	N	5	0	5
Atriplex hortensis	L.	Amaranthaceae	A	4	0	4
Atropa belladonna	L.	Solanaceae	N	1	0	1
Aubrieta deltoidea	(L.) DC.	Brassicaceae	A	14	0	14
Aucuba japonica	Thunb.	Garryaceae	A	4	0	4
Aurinia saxatilis	(L.) Desv.	Brassicaceae	A	11	0	11
Avena fatua	L.	Poaceae	N	0	1	1
Avena sativa	L.	Poaceae	A	0	4	4
Baptisia australis	(L.) R. Br.	Fabaceae	A	1	0	1
Barbarea vulgaris	R. Br.	Brassicaceae	N	3	0	3
Begonia Semperflorens Cultorum Group		Begoniaceae	A	10	0	10
Bellis perennis	L.	Asteraceae	N	8	51	59
Berberis julianae	C. K. Schneid.	Berberidaceae	A	2	0	2
Berberis thunbergii	DC.	Berberidaceae	A	5	0	5
Berberis verruculosa	Hemsl. & E. H. Wilson	Berberidaceae	A	2	0	2
Berberis vulgaris	L.	Berberidaceae	N	2	0	2
Bergenia crassifolia	(L.) Fritsch	Saxifragaceae	A	11	0	11
Beta vulgaris	L.	Amaranthaceae	A	34	0	34
Betula pendula	Roth	Betulaceae	N	3	5	8
Bidens ferulifolia	(Jacq.) DC.	Asteraceae	A	10	0	10
Bidens frondosa	L.	Asteraceae	A	0	1	1
Bletilla striata	(Thunb.) Rchb. f.	Orchidaceae	A	3	0	3
Borago officinalis	L.	Boraginaceae	A	25	0	25
Bougainvillea spectabilis	Willd.	Nyctaginaceae	A	1	0	1
Brachypodium pinnatum	(L.) P. Beauv.	Poaceae	N	1	0	1
Brachypodium sylvaticum	(Huds.) P. Beauv.	Poaceae	N	0	25	25
Brachyscome iberidifolia	Benth.	Asteraceae	A	1	0	1
Brassica napus	L.	Brassicaceae	A	0	1	1
Brassica oleracea	L.	Brassicaceae	A	43	0	43
Brassica rapa	L.	Brassicaceae	A	5	0	5
Briza media	L.	Poaceae	N	1	0	1
Bromus erectus	Huds.	Poaceae	N	2	0	2
Bromus hordeaceus	L.	Poaceae	N	1	3	4
Bromus inermis	Leyss.	Poaceae	A	0	1	1
Bromus ramosus	Huds.	Poaceae	N	0	1	1
Bromus sterilis	L.	Poaceae	N	0	11	11
Bromus tectorum	L.	Poaceae	N	0	1	1
Brugmansia suaveolens	(Humb. & Bonpl. ex Willd.) Bercht. & J. Presl	Solanaceae	A	3	0	3
Brunnera macrophylla	(Adams) I. M. Johnst.	Boraginaceae	A	10	0	10
Bryonia dioica	Jacq.	Cucurbitaceae	N	2	0	2
Buddleja alternifolia	Maxim.	Scrophulariaceae	A	1	0	1
Buddleja davidii	Franch.	Scrophulariaceae	A	10	4	14
Buglossoides purpurocaerulea	(L.) I. M. Johnst.	Boraginaceae	N	2	0	2
Buphthalmum salicifolium	L.	Asteraceae	N	3	0	3
Buxus sempervirens	L.	Buxaceae	N	13	0	13
Calamintha nepeta aggr.		Lamiaceae	N	3	0	3
Calendula arvensis	L.	Asteraceae	N	2	0	2
Calendula officinalis	L.	Asteraceae	A	32	0	32
Callistephus chinensis	(L.) Nees	Asteraceae	A	7	0	7
Caltha palustris	L.	Ranunculaceae	N	3	0	3
Calystegia sepium	(L.) R. Br.	Convolvulaceae	N	0	59	59
Camassia cusickii	S. Watson	Asparagaceae	A	1	0	1
Campanula carpatica	Jacq.	Campanulaceae	A	1	0	1
Campanula medium	L.	Campanulaceae	A	1	0	1
Campanula persicifolia	L.	Campanulaceae	N	12	0	12
Campanula portenschlagiana	Schult.	Campanulaceae	A	4	0	4
Campanula poscharskyana	Degen	Campanulaceae	A	16	0	16
Campanula rapunculoides	L.	Campanulaceae	N	2	0	2
Campanula rotundifolia	L.	Campanulaceae	N	2	0	2
Campanula trachelium	L.	Campanulaceae	N	8	1	9
Campsis radicans	(L.) Seem.	Bignoniaceae	A	6	0	6
Cannabis sativa	L.	Cannabaceae	A	4	0	4
Capsella bursa-pastoris	(L.) Medik.	Brassicaceae	N	0	1	1
Capsella rubella	Reut.	Brassicaceae	N	0	2	2
Capsicum annuum	L.	Solanaceae	A	29	0	29
Cardamine bulbifera	(L.) Crantz	Brassicaceae	N	1	0	1
Cardamine flexuosa aggr.		Brassicaceae	N	0	11	11
Cardamine hirsuta	L.	Brassicaceae	N	0	73	73
Cardamine impatiens	L.	Brassicaceae	N	0	2	2
Cardamine pentaphyllos	(L.) Crantz	Brassicaceae	N	0	1	1
Cardamine pratensis	L.	Brassicaceae	N	5	28	33
Cardaria draba	(L.) Desv.	Brassicaceae	A	0	1	1
Carex digitata	L.	Cyperaceae	N	0	1	1
Carex elata	All.	Cyperaceae	N	2	0	2
Carex flacca	Schreb.	Cyperaceae	N	0	5	5
Carex grayi	Carey	Cyperaceae	A	2	0	2
Carex hirta	L.	Cyperaceae	N	0	7	7
Carex morrowii	Boott	Cyperaceae	A	2	0	2
Carex muricata aggr.		Cyperaceae	N	0	20	20
Carex pendula	Huds.	Cyperaceae	N	2	2	4
Carex pilosa	Scop.	Cyperaceae	N	0	1	1
Carex remota	L.	Cyperaceae	N	0	2	2
Carex sylvatica	Huds.	Cyperaceae	N	0	31	31
Carlina vulgaris	L.	Asteraceae	N	1	0	1
Carpinus betulus	L.	Betulaceae	N	7	11	18
Caryopteris × clandonensis	Simmonds	Lamiaceae	A	7	0	7
Castanea sativa	Mill.	Fagaceae	N	1	0	1
Catalpa bignonioides	Walter	Bignoniaceae	A	1	0	1
Cedrus libani	A. Rich.	Pinaceae	A	1	0	1
Celtis australis	L.	Cannabaceae	N	1	0	1
Centaurea cyanus	L.	Asteraceae	N	7	0	7
Centaurea jacea	L.	Asteraceae	N	2	0	2
Centaurea montana	L.	Asteraceae	N	33	0	33
Centaurea pseudophrygia	C. A. Mey.	Asteraceae	N	1	0	1
Centaurea scabiosa	L.	Asteraceae	N	2	0	2
Centaurium erythraea	Rafn	Gentianaceae	N	1	0	1
Centranthus ruber	(L.) DC.	Caprifoliaceae	A	12	0	12
Cephalaria alpina	(L.) Roem. & Schult.	Caprifoliaceae	N	1	0	1
Cerastium brachypetalum	Pers.	Caryophyllaceae	N	0	2	2
Cerastium fontanum subsp. vulgare	(Hartm.) Greuter & Burdet	Caryophyllaceae	N	5	18	23
Cerastium glomeratum	Thuill.	Caryophyllaceae	N	0	2	2
Cerastium tomentosum	L.	Caryophyllaceae	A	9	0	9
Ceratophyllum demersum	L.	Ceratophyllaceae	N	1	0	1
Ceratostigma plumbaginoides	Bunge	Plumbaginaceae	A	7	0	7
Cercis siliquastrum	L.	Fabaceae	A	3	0	3
Cerinthe major	L.	Boraginaceae	A	1	0	1
Chaenomeles japonica	(Thunb.) Spach	Rosaceae	A	7	0	7
Chaenorrhinum minus	(L.) Lange	Plantaginaceae	N	0	18	18
Chaenostoma cordatum	(Thunb.) Benth.	Scrophulariaceae	A	1	0	1
Chamaecyparis lawsoniana	(A. Murray bis) Parl.	Cupressaceae	A	5	0	5
Chamaecyparis pisifera	(Siebold & Zucc.) Endl.	Cupressaceae	A	1	0	1
Chelidonium majus	L.	Papaveraceae	N	0	27	27
Chenopodium album aggr.		Amaranthaceae	N	0	5	5
Chenopodium bonus-henricus	L.	Amaranthaceae	N	1	0	1
Chenopodium giganteum	D. Don	Amaranthaceae	A	9	0	9
Chenopodium polyspermum	L.	Amaranthaceae	N	0	28	28
Chlorophytum comosum	(Thunb.) Jacques	Asparagaceae	A	1	0	1
Chrysanthemum × grandiflorum	Ramat.	Asteraceae	A	6	0	6
Cichorium endivia	L.	Asteraceae	A	13	0	13
Cichorium intybus	L.	Asteraceae	N	8	0	8
Circaea lutetiana	L.	Onagraceae	N	0	64	64
Cirsium arvense	(L.) Scop.	Asteraceae	N	0	5	5
Cirsium vulgare	(Savi) Ten.	Asteraceae	N	0	3	3
Cistus CV		Cistaceae	A	2	0	2
Citrus japonica	Thunb.	Rutaceae	A	4	0	4
Clematis alpina	(L.) Mill.	Ranunculaceae	N	3	0	3
Clematis CV		Ranunculaceae	A	16	0	16
Clematis montana	Buch.-Ham. ex DC.	Ranunculaceae	A	6	0	6
Clematis recta	L.	Ranunculaceae	N	1	0	1
Clematis vitalba	L.	Ranunculaceae	N	2	4	6
Cleome hassleriana	Schltdl.	Cleomaceae	A	4	0	4
Clinopodium vulgare	L.	Lamiaceae	N	1	0	1
Coffea arabica	L.	Rubiaceae	A	1	0	1
Consolida ajacis	(L.) Schur	Ranunculaceae	A	1	0	1
Convallaria majalis	L.	Asparagaceae	N	39	0	39
Convolvulus arvensis	L.	Convolvulaceae	N	0	7	7
Convolvulus tricolor	L.	Convolvulaceae	A	2	0	2
Conyza canadensis	(L.) Cronquist	Asteraceae	A	0	14	14
Coreopsis verticillata	L.	Asteraceae	A	0	1	1
Coriandrum sativum	L.	Apiaceae	A	2	0	2
Cornus alba	L.	Cornaceae	A	2	0	2
Cornus kousa	F. Buerger ex Hance	Cornaceae	A	2	0	2
Cornus mas	L.	Cornaceae	N	9	0	9
Cornus sanguinea	L.	Cornaceae	N	15	7	22
Cornus sericea	L.	Cornaceae	A	1	0	1
Cortaderia selloana	(Schult. & Schult. f.) Asch. & Graebn.	Poaceae	A	4	0	4
Corydalis cava	(L.) Schweigg. & Körte	Papaveraceae	N	0	9	9
Corydalis lutea	(L.) DC.	Papaveraceae	N	2	0	2
Corylopsis pauciflora	Siebold & Zucc.	Hamamelidaceae	A	5	0	5
Corylus avellana	L.	Betulaceae	N	14	9	23
Corylus maxima	Mill.	Betulaceae	A	5	0	5
Cosmos bipinnatus	Cav.	Asteraceae	A	20	0	20
Cosmos sulphureus	Cav.	Asteraceae	A	3	0	3
Cotinus coggygria	Scop.	Anacardiaceae	N	4	0	4
Cotoneaster bullatus	Bois	Rosaceae	A	1	0	1
Cotoneaster dammeri	C. K. Schneid.	Rosaceae	A	8	0	8
Cotoneaster dielsianus	E. Pritz.	Rosaceae	A	1	0	1
Cotoneaster divaricatus	Rehder & E. H. Wilson	Rosaceae	A	7	0	7
Cotoneaster horizontalis	Decne.	Rosaceae	A	4	0	4
Cotoneaster salicifolius	Franch.	Rosaceae	A	2	0	2
Crataegus monogyna aggr.		Rosaceae	N	8	5	13
Crepis biennis	L.	Asteraceae	N	6	4	10
Crepis capillaris	Wallr.	Asteraceae	N	0	20	20
Crocosmia × crocosmiiflora	(Lemoine) N. E. Br.	Iridaceae	A	27	0	27
Cucumis melo	L.	Cucurbitaceae	A	6	0	6
Cucumis sativus	L.	Cucurbitaceae	A	33	0	33
Cucurbita maxima	Duchesne	Cucurbitaceae	A	14	0	14
Cucurbita pepo	L.	Cucurbitaceae	A	44	0	44
Cuphea ignea	A. DC.	Lythraceae	A	1	0	1
Cyclamen coum	Mill.	Primulaceae	A	1	0	1
Cydonia oblonga	Mill.	Rosaceae	A	15	0	15
Cymbalaria muralis	G. Gaertn. & al.	Plantaginaceae	N	6	1	7
Cynara cardunculus subsp. scolymus	(L.) Beger	Asteraceae	A	12	0	12
Cynodon dactylon	(L.) Pers.	Poaceae	N	0	1	1
Cynosurus cristatus	L.	Poaceae	N	2	0	2
Cyperus involucratus	Rottb.	Cyperaceae	A	1	0	1
Cytisus × praecox	Wheeler ex. Bean	Fabaceae	A	1	0	1
Dactylis glomerata	L.	Poaceae	N	11	18	29
Dahlia CV		Asteraceae	A	14	0	14
Dahlia merckii	Lehm.	Asteraceae	A	1	0	1
Daphne mezereum	L.	Thymelaeaceae	N	2	0	2
Datura innoxia	Mill.	Solanaceae	A	1	0	1
Datura stramonium	L.	Solanaceae	A	1	0	1
Daucus carota subsp. carota	L.	Apiaceae	N	12	4	16
Daucus carota subsp. sativus	(Hoffm.) Arcang.	Apiaceae	N	17	0	17
Delphinium × cultorum	Voss.	Ranunculaceae	A	6	0	6
Deschampsia cespitosa	(L.) P. Beauv.	Poaceae	N	0	4	4
Deutzia gracilis	Siebold & Zucc.	Hydrangeaceae	A	5	0	5
Deutzia scabra	Thunb.	Hydrangeaceae	A	2	0	2
Dianthus armeria	L.	Caryophyllaceae	N	0	6	6
Dianthus barbatus	L.	Caryophyllaceae	A	13	0	13
Dianthus carthusianorum	L.	Caryophyllaceae	N	4	0	4
Dianthus chinensis	L.	Caryophyllaceae	A	8	0	8
Dianthus deltoides	L.	Caryophyllaceae	N	3	0	3
Dianthus gratianopolitanus	Vill.	Caryophyllaceae	N	10	0	10
Dicentra spectabilis	(L.) Fukuhara	Papaveraceae	A	15	0	15
Dictamnus albus	L.	Rutaceae	N	1	0	1
Digitalis grandiflora	Mill.	Plantaginaceae	N	2	0	2
Digitalis lutea	L.	Plantaginaceae	N	4	0	4
Digitalis purpurea	L.	Plantaginaceae	A	20	0	20
Digitaria sanguinalis	(L.) Scop.	Poaceae	N	0	53	53
Dionaea muscipula	J.Ellis	Droseraceae	A	1	0	1
Diospyros lotus	L.	Ebenaceae	A	1	0	1
Diplotaxis tenuifolia	(L.) DC.	Brassicaceae	N	16	0	16
Dipsacus fullonum	L.	Caprifoliaceae	N	11	0	11
Dipsacus laciniatus	L.	Caprifoliaceae	N	1	0	1
Dipsacus pilosus	L.	Caprifoliaceae	N	0	1	1
Doronicum orientale	Hoffm.	Asteraceae	A	5	0	5
Dracunculus vulgaris	Schott	Araceae	A	4	0	4
Dryopteris cycadina	(Franch. & Sav.) C. Chr.	Dryopteridaceae	A	1	0	1
Dryopteris filix-mas	(L.) Schott	Dryopteridaceae	N	48	0	48
Duchesnea indica	(Andrews) Focke	Rosaceae	A	0	24	24
Echeveria elegans	Rose	Crassulaceae	A	1	0	1
Echinacea purpurea	(L.) Moench	Asteraceae	A	21	0	21
Echinochloa crus-galli	(L.) P. Beauv.	Poaceae	N	0	4	4
Echinops sphaerocephalus	L.	Asteraceae	N	5	0	5
Echium vulgare	L.	Boraginaceae	N	5	0	5
Eichhornia crassipes	(Mart.) Solms	Pontederiaceae	A	1	0	1
Elaeagnus × submacrophylla	Servett.	Elaeagnaceae	A	1	0	1
Eleocharis palustris aggr.		Cyperaceae	N	1	0	1
Elodea canadensis	Michx.	Hydrocharitaceae	A	1	0	1
Elymus caninus	(L.) L.	Poaceae	N	0	2	2
Elymus repens	(L.) Gould	Poaceae	N	0	22	22
Epilobium angustifolium	L.	Onagraceae	N	5	0	5
Epilobium dodonaei	Vill.	Onagraceae	N	2	0	2
Epilobium hirsutum	L.	Onagraceae	N	0	10	10
Epilobium montanum	L.	Onagraceae	N	0	32	32
Epilobium parviflorum	Schreb.	Onagraceae	N	0	39	39
Epilobium roseum	Schreb.	Onagraceae	N	0	37	37
Epilobium tetragonum	L.	Onagraceae	N	0	5	5
Epimedium pinnatum	Fisch.	Berberidaceae	A	5	0	5
Equisetum arvense	L.	Equisetaceae	N	0	15	15
Equisetum telmateia	Ehrh.	Equisetaceae	N	0	1	1
Eragrostis minor	Host	Poaceae	N	0	6	6
Eranthis hyemalis	(L.) Salisb.	Ranunculaceae	N	19	0	19
Erica carnea	L.	Ericaceae	N	3	0	3
Erigeron annuus	(L.) Desf.	Asteraceae	A	0	17	17
Erigeron karvinskianus	DC.	Asteraceae	A	1	0	1
Erinus alpinus	L.	Plantaginaceae	N	2	0	2
Erodium × hybridum	Sünd.	Geraniaceae	A	1	0	1
Erodium × variabile	A. C. Leslie	Geraniaceae	A	1	0	1
Erophila verna aggr.		Brassicaceae	N	0	1	1
Eruca sativa	(L.) Cav.	Brassicaceae	A	9	0	9
Erysimum cheiri	(L.) Crantz	Brassicaceae	A	8	0	8
Eschscholzia californica	Cham.	Papaveraceae	A	4	0	4
Euonymus europaeus	L.	Celastraceae	N	8	0	8
Euonymus fortunei	(Turcz.) Hand.-Mazz.	Celastraceae	A	7	0	7
Euonymus japonicus	Thunb.	Celastraceae	A	6	0	6
Eupatorium cannabinum	L.	Asteraceae	N	2	1	3
Euphorbia amygdaloides	L.	Euphorbiaceae	N	5	0	5
Euphorbia characias	L.	Euphorbiaceae	A	1	0	1
Euphorbia cyparissias	L.	Euphorbiaceae	N	4	0	4
Euphorbia dulcis	L.	Euphorbiaceae	N	0	2	2
Euphorbia epithymoides	L.	Euphorbiaceae	A	1	0	1
Euphorbia helioscopia	L.	Euphorbiaceae	N	0	2	2
Euphorbia lathyris	L.	Euphorbiaceae	A	18	0	18
Euphorbia maculata aggr.		Euphorbiaceae	A	0	7	7
Euphorbia myrsinites	L.	Euphorbiaceae	A	1	0	1
Euphorbia palustris	L.	Euphorbiaceae	N	1	0	1
Euphorbia peplus	L.	Euphorbiaceae	N	0	62	62
Euphorbia verrucosa	L.	Euphorbiaceae	N	1	0	1
Eutrema japonicum	(Miq.) Koidz.	Brassicaceae	A	1	0	1
Fagopyrum esculentum	Moench	Polygonaceae	A	5	0	5
Fagus sylvatica	L.	Fagaceae	N	5	1	6
Fallopia aubertii	(L.Henry) Holub	Polygonaceae	A	2	0	2
Festuca arundinacea	Schreb.	Poaceae	N	24	0	24
Festuca cinerea	Vill.	Poaceae	A	1	0	1
Festuca gigantea	(L.) Vill.	Poaceae	N	0	1	1
Festuca ovina aggr.		Poaceae	N	0	4	4
Festuca rubra aggr.		Poaceae	N	67	0	67
Ficus carica	L.	Moraceae	N	17	0	17
Filipendula ulmaria	(L.) Maxim.	Rosaceae	N	0	2	2
Filipendula vulgaris	Moench	Rosaceae	N	2	0	2
Foeniculum vulgare	Mill.	Apiaceae	A	26	0	26
Forsythia × intermedia	Zabel	Oleaceae	A	27	0	27
Fragaria × ananassa	(Weston) Rozier	Rosaceae	A	31	0	31
Fragaria moschata	Duchesne	Rosaceae	N	1	0	1
Fragaria vesca	L.	Rosaceae	N	0	66	66
Frangula alnus	Mill.	Rhamnaceae	N	2	0	2
Fraxinus excelsior	L.	Oleaceae	N	0	24	24
Fritillaria imperialis	L.	Liliaceae	A	4	0	4
Fritillaria meleagris	L.	Liliaceae	N	2	0	2
Fuchsia magellanica	Lam.	Onagraceae	A	21	0	21
Fumaria officinalis	L.	Papaveraceae	N	0	2	2
Gaillardia × grandiflora	Hort. ex Van Houtte	Asteraceae	A	1	0	1
Galanthus elwesii	Hook. f.	Amaryllidaceae	A	4	0	4
Galanthus nivalis	L.	Amaryllidaceae	N	43	0	43
Galeopsis tetrahit	L.	Lamiaceae	N	0	16	16
Galinsoga quadriradiata	Ruiz & Pav.	Asteraceae	A	0	8	8
Galium aparine	L.	Rubiaceae	N	0	6	6
Galium mollugo aggr.		Rubiaceae	N	5	13	18
Galium odoratum	(L.) Scop.	Rubiaceae	N	1	12	13
Galium verum	L.	Rubiaceae	N	4	0	4
Gaura lindheimeri	Engelm. & A.Gray	Onagraceae	A	6	0	6
Gazania CV		Asteraceae	A	2	0	2
Gentiana acaulis	L.	Gentianaceae	N	7	0	7
Geranium × cantabrigiense	P. F. Yeo	Geraniaceae	A	3	0	3
Geranium × magnificum	Hyl.	Geraniaceae	A	16	0	16
Geranium × oxonianum	P. F. Yeo	Geraniaceae	A	2	0	2
Geranium dissectum	L.	Geraniaceae	N	0	1	1
Geranium himalayense	Klotzsch	Geraniaceae	A	1	0	1
Geranium macrorrhizum	L.	Geraniaceae	A	11	0	11
Geranium nodosum	L.	Geraniaceae	N	1	0	1
Geranium palustre	L.	Geraniaceae	N	1	0	1
Geranium phaeum	L.	Geraniaceae	N	3	0	3
Geranium pratense	L.	Geraniaceae	N	9	0	9
Geranium pyrenaicum	Burm. f.	Geraniaceae	N	0	13	13
Geranium renardii	Trautv.	Geraniaceae	A	4	0	4
Geranium robertianum subsp. purpureum	Vill.	Geraniaceae	N	0	1	1
Geranium robertianum subsp. robertianum	L.	Geraniaceae	N	0	50	50
Geranium sanguineum	L.	Geraniaceae	N	10	0	10
Geranium sylvaticum	L.	Geraniaceae	N	2	0	2
Geum coccineum	Sibth. & Sm.	Rosaceae	A	3	0	3
Geum rivale	L.	Rosaceae	N	0	2	2
Geum urbanum	L.	Rosaceae	N	0	72	72
Ginkgo biloba	L.	Ginkgoaceae	A	4	0	4
Gladiolus × hortulanus	L.H.Bailey	Iridaceae	A	21	0	21
Gladiolus callianthus	Kelway	Iridaceae	A	1	0	1
Gladiolus communis	L.	Iridaceae	A	1	0	1
Glebionis segetum	(L.) Fourr.	Asteraceae	A	2	0	2
Glechoma hederacea	L.	Lamiaceae	N	0	50	50
Gleditsia triacanthos	L.	Fabaceae	A	1	0	1
Glycine max	(L.) Merr.	Fabaceae	A	3	0	3
Gynostemma pentaphyllum	(Thunb.) Makino	Cucurbitaceae	A	2	0	2
Gypsophila paniculata	L.	Caryophyllaceae	A	2	0	2
Gypsophila repens	L.	Caryophyllaceae	N	2	0	2
Hamamelis mollis	Oliv. ex F. B. Forbes & Hemsl.	Hamamelidaceae	A	2	0	2
Hebe odora	Cockayne	Scrophulariaceae	A	4	0	4
Hedera helix	L.	Araliaceae	N	43	5	48
Helenium autumnale	L.	Asteraceae	A	4	0	4
Helianthemum nummularium	Dun.	Cistaceae	N	1	0	1
Helianthus annuus	L.	Asteraceae	A	31	0	31
Helianthus pauciflorus	Nutt.	Asteraceae	A	5	0	5
Helianthus tuberosus	L.	Asteraceae	A	13	0	13
Helichrysum bracteatum	(Venten.) Willd.	Asteraceae	A	1	0	1
Helichrysum italicum	(Roth) G. Don	Asteraceae	A	6	0	6
Helictotrichon pubescens	(Huds.) Pilg.	Poaceae	N	2	0	2
Heliopsis helianthoides	(L.) Sweet	Asteraceae	A	2	0	2
Helleborus foetidus	L.	Ranunculaceae	N	4	0	4
Helleborus niger	L.	Ranunculaceae	N	11	0	11
Helleborus orientalis	Lam.	Ranunculaceae	A	32	0	32
Hemerocallis fulva	(L.) L.	Asphodelaceae	A	15	0	15
Hemerocallis lilioasphodelus	L.	Asphodelaceae	A	1	0	1
Hepatica nobilis	Schreb.	Ranunculaceae	N	2	0	2
Heracleum sphondylium	L.	Apiaceae	N	2	2	4
Herniaria hirsuta	L.	Caryophyllaceae	N	0	1	1
Hesperis matronalis	L.	Brassicaceae	A	4	0	4
Heuchera americana	L.	Saxifragaceae	A	14	0	14
Hibiscus syriacus	L.	Malvaceae	A	34	0	34
Hibiscus trionum	L.	Malvaceae	A	1	0	1
Hieracium aurantiacum	L.	Asteraceae	N	17	0	17
Hieracium lachenalii	C. C. Gmel.	Asteraceae	N	0	1	1
Hieracium murorum aggr.		Asteraceae	N	0	2	2
Hieracium pilosella	L.	Asteraceae	N	3	4	7
Hieracium piloselloides	Vill.	Asteraceae	N	1	0	1
Hippocrepis emerus	(L.) Lassen	Fabaceae	N	2	0	2
Hippophaë rhamnoides	L.	Elaeagnaceae	N	5	0	5
Hippuris vulgaris	L.	Plantaginaceae	N	2	0	2
Holcus lanatus	L.	Poaceae	N	5	12	17
Hordeum murinum	L.	Poaceae	N	0	1	1
Hordeum vulgare aggr.		Poaceae	A	0	1	1
Hosta CV		Asparagaceae	A	16	0	16
Humulus lupulus	L.	Cannabaceae	N	4	4	8
Hyacinthoides non-scripta	(L.) Rothm.	Asparagaceae	A	42	0	42
Hyacinthus orientalis	L.	Asparagaceae	A	41	0	41
Hydrangea aspera	D. Don	Hydrangeaceae	A	1	0	1
Hydrangea macrophylla	(Thunb.) Ser.	Hydrangeaceae	A	32	0	32
Hydrangea paniculata	Siebold	Hydrangeaceae	A	2	0	2
Hydrangea petiolaris	Siebold & Zucc.	Hydrangeaceae	A	10	0	10
Hydrangea quercifolia	W. Bartram	Hydrangeaceae	A	1	0	1
Hypericum androsaemum	L.	Hypericaceae	N	2	0	2
Hypericum calycinum	L.	Hypericaceae	A	3	0	3
Hypericum perforatum	L.	Hypericaceae	N	8	7	15
Hypericum tetrapterum	Fr.	Hypericaceae	N	0	2	2
Hypochaeris radicata	L.	Asteraceae	N	4	18	22
Hyssopus officinalis	L.	Lamiaceae	N	6	0	6
Iberis sempervirens	L.	Brassicaceae	A	23	0	23
Iberis umbellata	L.	Brassicaceae	A	3	0	3
Ilex aquifolium	L.	Aquifoliaceae	N	18	1	19
Impatiens balfourii	Hook. f.	Balsaminaceae	A	0	1	1
Impatiens balsamina	L.	Balsaminaceae	A	1	0	1
Impatiens parviflora	DC.	Balsaminaceae	A	0	1	1
Impatiens walleriana	Hook. f.	Balsaminaceae	A	3	0	3
Inula conyzae	(Griess.) Meikle	Asteraceae	N	0	1	1
Inula ensifolia	L.	Asteraceae	A	1	0	1
Inula helenium	L.	Asteraceae	A	4	0	4
Ipomoea × multifida	(Raf.) Shinners	Convolvulaceae	A	1	0	1
Ipomoea batatas	(L.) Lam.	Convolvulaceae	A	1	0	1
Ipomoea purpurea	(L.) Roth	Convolvulaceae	A	11	0	11
Iris × germanica	L.	Iridaceae	N	32	0	32
Iris × hollandica	hort.	Iridaceae	A	3	0	3
Iris foetidissima	L.	Iridaceae	A	2	0	2
Iris pseudacorus	L.	Iridaceae	N	4	1	5
Iris sibirica	L.	Iridaceae	N	6	0	6
Isatis tinctoria	L.	Brassicaceae	N	1	0	1
Ismelia carinata	(Schousb.) Sch.Bip.	Asteraceae	A	3	0	3
Jasminum nudiflorum	Lindl.	Oleaceae	A	8	0	8
Juglans regia	L.	Juglandaceae	N	6	6	12
Juncus effusus	L.	Juncaceae	N	0	1	1
Juncus inflexus	L.	Juncaceae	N	0	2	2
Juncus subnodulosus	Schrank	Juncaceae	N	0	1	1
Juncus tenuis	Willd.	Juncaceae	A	0	1	1
Juniperus chinensis	L.	Cupressaceae	A	2	0	2
Juniperus communis	L.	Cupressaceae	N	1	0	1
Kerria japonica	(L.) DC.	Rosaceae	A	12	0	12
Kickxia spuria	(L.) Dumort.	Plantaginaceae	N	0	2	2
Knautia arvensis	(L.) Coult.	Caprifoliaceae	N	4	0	4
Knautia dipsacifolia	Kreutzer	Caprifoliaceae	N	2	0	2
Kniphofia CV		Liliaceae	A	1	0	1
Kolkwitzia amabilis	Graebn.	Caprifoliaceae	A	8	0	8
Laburnum anagyroides	Medik.	Fabaceae	N	1	0	1
Lactuca sativa	L.	Asteraceae	A	29	0	29
Lactuca serriola	L.	Asteraceae	N	0	2	2
Lamium album	L.	Lamiaceae	N	1	0	1
Lamium galeobdolon subsp. argentatum	(Smejkal) J. Duvign.	Lamiaceae	A	19	0	19
Lamium maculatum	(L.) L.	Lamiaceae	N	1	3	4
Lamium purpureum	L.	Lamiaceae	N	0	6	6
Lampranthus spectabilis	(Haw.) N. E. Br.	Aizoaceae	A	1	0	1
Lantana camara	L.	Verbenaceae	A	2	0	2
Lapsana communis	L.	Asteraceae	N	0	35	35
Larix decidua	Mill.	Pinaceae	N	1	0	1
Lathyrus latifolius	L.	Fabaceae	N	9	0	9
Lathyrus pratensis	L.	Fabaceae	N	2	2	4
Lathyrus vernus	(L.) Bernh.	Fabaceae	N	3	0	3
Laurus nobilis	L.	Lauraceae	N	9	0	9
Lavandula × intermedia	Loisel.	Lamiaceae	A	53	0	53
Lavandula stoechas	L.	Lamiaceae	A	9	0	9
Lavatera × clementii	Cheek	Malvaceae	A	1	0	1
Lavatera trimestris	L.	Malvaceae	A	7	0	7
Lemna minor	L.	Araceae	N	4	0	4
Lemna trisulca	L.	Araceae	N	1	0	1
Leontodon hispidus	L.	Asteraceae	N	4	2	6
Leontopodium alpinum	(Cass.) Greuter	Asteraceae	N	3	0	3
Leonurus cardiaca	L.	Lamiaceae	N	2	0	2
Lepidium latifolium	L.	Brassicaceae	A	1	0	1
Lepidium sativum	L.	Brassicaceae	A	1	0	1
Lepidium virginicum	L.	Brassicaceae	A	0	1	1
Leucanthemum vulgare aggr.		Asteraceae	N	15	7	22
Leucojum aestivum	L.	Amaryllidaceae	N	4	0	4
Leucojum vernum	L.	Amaryllidaceae	N	5	0	5
Levisticum officinale	W. D. J. Koch	Apiaceae	A	22	0	22
Liatris spicata	(L.) Willd.	Asteraceae	A	1	0	1
Ligularia przewalskii	(Maxim.) Diels	Asteraceae	A	6	0	6
Ligustrum japonicum	Thunb.	Oleaceae	A	2	0	2
Ligustrum ovalifolium	Hausskn.	Oleaceae	A	14	0	14
Ligustrum vulgare	L.	Oleaceae	N	16	1	17
Lilium CV		Liliaceae	A	11	0	11
Lilium martagon	L.	Liliaceae	N	2	0	2
Limonium platyphyllum	Lincz.	Plumbaginaceae	A	2	0	2
Limonium sinuatum	(L.) Mill.	Plumbaginaceae	A	1	0	1
Linaria maroccana	Hook. f.	Plantaginaceae	A	4	0	4
Linaria purpurea	(L.) Mill.	Plantaginaceae	A	19	0	19
Linaria vulgaris	Mill.	Plantaginaceae	N	0	6	6
Linum grandiflorum	Desf.	Linaceae	A	2	0	2
Linum usitatissimum	L.	Linaceae	A	6	0	6
Liriodendron tulipifera	L.	Magnoliaceae	A	3	0	3
Lobelia erinus	L.	Campanulaceae	A	2	0	2
Lobelia siphilitica	L.	Campanulaceae	A	2	0	2
Lobularia maritima	(L.) Desv.	Brassicaceae	A	8	0	8
Lolium perenne	L.	Poaceae	N	70	0	70
Lonicera × purpusii	Rehder	Caprifoliaceae	A	3	0	3
Lonicera × tellmanniana	Magyar ex H. L. Späth	Caprifoliaceae	A	5	0	5
Lonicera caprifolium	L.	Caprifoliaceae	N	7	0	7
Lonicera henryi	Hemsl.	Caprifoliaceae	A	3	0	3
Lonicera japonica	Thunb.	Caprifoliaceae	A	5	0	5
Lonicera kamtschatica	Pojark.	Caprifoliaceae	A	3	0	3
Lonicera periclymenum	L.	Caprifoliaceae	N	6	0	6
Lonicera pileata	Oliv.	Caprifoliaceae	A	21	0	21
Lonicera xylosteum	L.	Caprifoliaceae	N	5	0	5
Lotus corniculatus	L.	Fabaceae	N	3	1	4
Lotus tetragonolobus	L.	Fabaceae	A	1	0	1
Lunaria annua	L.	Brassicaceae	A	3	0	3
Lunaria rediviva	L.	Brassicaceae	N	4	0	4
Lupinus polyphyllus	Lindl.	Fabaceae	A	9	0	9
Luzula campestris	(L.) DC.	Juncaceae	N	0	1	1
Luzula nivea	(L.) DC.	Juncaceae	N	3	0	3
Luzula sylvatica	(Huds.) Gaudin	Juncaceae	N	2	0	2
Lycianthes rantonnetii	(Carrière ex Lesc.) Bitter	Solanaceae	A	2	0	2
Lycium barbarum	L.	Solanaceae	A	2	0	2
Lycopersicon esculentum	Mill.	Solanaceae	A	49	0	49
Lysimachia clethroides	Duby	Primulaceae	A	3	0	3
Lysimachia nummularia	L.	Primulaceae	N	0	11	11
Lysimachia punctata	L.	Primulaceae	A	17	0	17
Lysimachia vulgaris	L.	Primulaceae	N	5	0	5
Lythrum salicaria	L.	Lythraceae	N	15	3	18
Magnolia × soulangeana	Soul.-Bod.	Magnoliaceae	A	4	0	4
Mahonia aquifolium	(Pursh) Nutt.	Berberidaceae	A	9	0	9
Malus pumila	Mill.	Rosaceae	A	43	0	43
Malva alcea	L.	Malvaceae	N	6	0	6
Malva moschata	L.	Malvaceae	N	10	1	11
Malva neglecta	Wallr.	Malvaceae	N	0	4	4
Malva sylvestris	L.	Malvaceae	N	18	0	18
Malva verticillata	L.	Malvaceae	A	1	0	1
Mandevilla splendens	(Hook. f.) Woodson	Apocynaceae	A	4	0	4
Matricaria chamomilla	L.	Asteraceae	N	1	6	7
Matricaria discoidea	DC.	Asteraceae	A	0	1	1
Matteuccia struthiopteris	(L.) Tod.	Onocleaceae	N	9	0	9
Matthiola incana	(L.) R. Br.	Brassicaceae	A	3	0	3
Meconopsis cambrica	(L.) Vig.	Papaveraceae	A	5	0	5
Medicago lupulina	L.	Fabaceae	N	0	19	19
Medicago sativa	L.	Fabaceae	N	2	2	4
Melampyrum arvense	L.	Orobanchaceae	N	1	0	1
Melica nutans	L.	Poaceae	N	2	2	4
Melilotus albus	Medik.	Fabaceae	N	0	1	1
Melilotus officinalis	(L.) Pall.	Fabaceae	N	2	0	2
Melissa officinalis	L.	Lamiaceae	N	42	0	42
Melittis melissophyllum	L.	Lamiaceae	N	2	0	2
Melothria scabra	Naudin	Cucurbitaceae	A	3	0	3
Mentha × piperita	L.	Lamiaceae	A	50	0	50
Mentha aquatica	L.	Lamiaceae	N	2	0	2
Mentha longifolia	(L.) Huds.	Lamiaceae	N	1	3	4
Mentha spicata	L.	Lamiaceae	A	18	0	18
Mentha suaveolens	Ehrh.	Lamiaceae	N	13	0	13
Menyanthes trifoliata	L.	Menyanthaceae	N	4	0	4
Mercurialis perennis	L.	Euphorbiaceae	N	0	2	2
Mespilus germanica	L.	Rosaceae	N	1	0	1
Mirabilis jalapa	L.	Nyctaginaceae	A	1	0	1
Miscanthus sinensis	Andersson	Poaceae	A	5	0	5
Moehringia trinervia	(L.) Clairv.	Caryophyllaceae	N	0	1	1
Molinia caerulea	(L.) Moench	Poaceae	N	1	0	1
Monarda didyma	L.	Lamiaceae	A	11	0	11
Morus nigra	L.	Moraceae	A	3	0	3
Muehlenbeckia complexa	Meisn.	Polygonaceae	A	1	0	1
Musa basjoo	Siebold & Zucc. ex Iinuma	Musaceae	A	2	0	2
Muscari armeniacum	Baker	Asparagaceae	A	33	0	33
Mycelis muralis	(L.) Dumort.	Asteraceae	N	0	6	6
Myosotis arvensis	Hill	Boraginaceae	N	0	10	10
Myosotis sylvatica	Hoffm.	Boraginaceae	N	31	0	31
Myriophyllum spicatum	L.	Haloragaceae	N	1	0	1
Myrrhis odorata	(L.) Scop.	Apiaceae	N	1	0	1
Narcissus cyclamineus	DC.	Amaryllidaceae	A	39	0	39
Narcissus jonquilla	L.	Amaryllidaceae	A	1	0	1
Narcissus poëticus	L.	Amaryllidaceae	N	1	0	1
Narcissus pseudonarcissus	L.	Amaryllidaceae	N	57	0	57
Narcissus tazetta	L.	Amaryllidaceae	A	4	0	4
Narthecium ossifragum	(L.) Huds.	Nartheciaceae	A	1	0	1
Nepeta × faasenii	Stearn	Lamiaceae	A	9	0	9
Nerium oleander	L.	Apocynaceae	A	6	0	6
Nicotiana × sanderae	W. Watson	Solanaceae	A	1	0	1
Nicotiana tabacum	L.	Solanaceae	A	1	0	1
Nigella damascena	L.	Ranunculaceae	A	20	0	20
Nymphaea alba	L.	Nymphaeaceae	N	10	0	10
Nymphoides peltata	(S. G. Gmel.) Kuntze	Gentianaceae	N	1	0	1
Ocimum basilicum	L.	Lamiaceae	A	29	0	29
Ocimum tenuiflorum	L.	Lamiaceae	A	2	0	2
Oenothera biennis aggr.		Onagraceae	A	1	32	33
Oenothera fruticosa	L.	Onagraceae	A	1	0	1
Oenothera parviflora aggr.		Onagraceae	A	0	1	1
Olea europaea	L.	Oleaceae	A	4	0	4
Onobrychis viciifolia	Scop.	Fabaceae	A	2	0	2
Onoclea sensibilis	L.	Onocleaceae	A	1	0	1
Opuntia humifusa	(Raf.) Raf.	Cactaceae	A	1	0	1
Opuntia phaeacantha	Engelm.	Cactaceae	A	3	0	3
Origanum majorana	L.	Lamiaceae	A	2	0	2
Origanum vulgare	L.	Lamiaceae	N	41	1	42
Orlaya grandiflora	(L.) Hoffm.	Apiaceae	N	1	0	1
Ornithogalum umbellatum	L.	Asparagaceae	N	2	0	2
Orobanche hederae	Duby	Orobanchaceae	N	0	1	1
Osmanthus heterophyllus	(G.Don) P. S. Green	Oleaceae	A	1	0	1
Osmunda regalis	L.	Osmundaceae	N	1	0	1
Oxalis corniculata	L.	Oxalidaceae	N	0	57	57
Oxalis debilis	Kunth	Oxalidaceae	A	5	0	5
Oxalis stricta	L.	Oxalidaceae	A	0	48	48
Oxalis tetraphylla	Cav.	Oxalidaceae	A	5	0	5
Oxalis triangularis	A. St.-Hil.	Oxalidaceae	A	3	0	3
Pachysandra terminalis	Siebold & Zucc.	Buxaceae	A	1	0	1
Paeonia × suffruticosa	Andrews	Paeoniaceae	A	7	0	7
Paeonia lactiflora	Pall.	Paeoniaceae	A	41	0	41
Paeonia lutea	Franch.	Paeoniaceae	A	1	0	1
Paeonia officinalis	L.	Paeoniaceae	N	9	0	9
Panicum capillare aggr.		Poaceae	A	0	10	10
Panicum miliaceum	L.	Poaceae	A	0	1	1
Panicum virgatum	L.	Poaceae	A	1	0	1
Papaver croceum	L.	Papaveraceae	A	1	0	1
Papaver dubium	L.	Papaveraceae	N	0	1	1
Papaver orientale	L.	Papaveraceae	A	13	0	13
Papaver rhoeas	L.	Papaveraceae	N	6	0	6
Papaver somniferum	L.	Papaveraceae	A	9	0	9
Parrotia persica	C.A. Mey.	Hamamelidaceae	A	2	0	2
Parthenocissus quinquefolia aggr.		Vitaceae	A	16	0	16
Parthenocissus tricuspidata	(Siebold & Zucc.) Planch.	Vitaceae	A	7	0	7
Passiflora caerulea	L.	Passifloraceae	A	4	0	4
Pastinaca sativa	L.	Apiaceae	N	4	0	4
Pelargonium CV		Geraniaceae	A	21	0	21
Pennisetum alopecuroides	(L.) Spreng.	Poaceae	A	7	0	7
Perilla frutescens	(L.) Britton	Lamiaceae	A	1	0	1
Perovskia atriplicifolia	Benth.	Lamiaceae	A	4	0	4
Persea americana	Mill.	Lauraceae	A	1	0	1
Petasites albus	(L.) Gaertn.	Asteraceae	N	1	0	1
Petrorhagia saxifraga	(L.) Link	Caryophyllaceae	N	2	0	2
Petroselinum crispum	(Mill.) Fuss	Apiaceae	A	25	0	25
Petunia × atkinsiana	D. Don ex W. H. Baxter	Solanaceae	A	12	0	12
Phacelia campanularia	A. Gray	Boraginaceae	A	1	0	1
Phacelia tanacetifolia	Benth.	Boraginaceae	A	11	0	11
Phalaris arundinacea	L.	Poaceae	N	2	0	2
Phalaris canariensis	L.	Poaceae	A	0	1	1
Phaseolus vulgaris	L.	Fabaceae	A	36	0	36
Philadelphus coronarius	L.	Hydrangeaceae	A	18	0	18
Phleum pratense	L.	Poaceae	N	1	2	3
Phlomis fruticosa	L.	Lamiaceae	A	1	0	1
Phlomis russeliana	Benth.	Lamiaceae	A	2	0	2
Phlox paniculata	L.	Polemoniaceae	A	32	0	32
Phlox subulata	L.	Polemoniaceae	A	10	0	10
Phoenix roebelenii	O'Brien	Arecaceae	A	2	0	2
Phyllitis scolopendrium	(L.) Newman	Aspleniaceae	N	8	0	8
Phyllostachys aurea	Rivière & C. Rivière	Poaceae	A	1	0	1
Physalis alkekengi	L.	Solanaceae	A	5	0	5
Physalis peruviana	L.	Solanaceae	A	13	0	13
Physalis philadelphica	Lam.	Solanaceae	A	2	0	2
Physostegia virginiana	(L.) Benth.	Lamiaceae	A	5	0	5
Picea abies	(L.) H. Karst.	Pinaceae	N	10	0	10
Picea glauca var. albertiana	S. Br.	Pinaceae	A	7	0	7
Picea omorika	(Pancic) Purk.	Pinaceae	A	4	0	4
Picea pungens	Engelm.	Pinaceae	A	2	0	2
Picris hieracioides	L.	Asteraceae	N	0	1	1
Pieris japonica	(Thunb.) D. Don ex G. Don	Ericaceae	A	1	0	1
Pimpinella major	(L.) Huds.	Apiaceae	N	1	0	1
Pimpinella saxifraga	L.	Apiaceae	N	2	0	2
Pinus cembra	L.	Pinaceae	N	1	0	1
Pinus mugo	Turra	Pinaceae	N	10	0	10
Pinus strobus	L.	Pinaceae	A	1	0	1
Pinus sylvestris	L.	Pinaceae	N	3	0	3
Pisum sativum	L.	Fabaceae	N	6	0	6
Plantago coronopus	L.	Plantaginaceae	A	1	0	1
Plantago lanceolata	L.	Plantaginaceae	N	10	28	38
Plantago major subsp. intermedia	(Gilib.) Lange	Plantaginaceae	N	0	1	1
Plantago major subsp. major	L.	Plantaginaceae	N	0	43	43
Plantago media	L.	Plantaginaceae	N	3	1	4
Platycodon grandiflorus	(Jacq.) A. DC.	Campanulaceae	A	3	0	3
Plumbago auriculata	Lam.	Plumbaginaceae	A	1	0	1
Poa annua	L.	Poaceae	N	0	66	66
Poa compressa	L.	Poaceae	N	0	2	2
Poa nemoralis	L.	Poaceae	N	0	4	4
Poa pratensis	L.	Poaceae	N	31	0	31
Poa trivialis	L.	Poaceae	N	10	40	50
Polemonium caeruleum	L.	Polemoniaceae	N	3	0	3
Polygonatum multiflorum	(L.) All.	Asparagaceae	N	3	14	17
Polygonum aviculare aggr.		Polygonaceae	N	0	7	7
Polygonum bistorta	L.	Polygonaceae	N	1	0	1
Polygonum persicaria	L.	Polygonaceae	N	0	13	13
Polystichum setiferum	(Forssk.) Woyn.	Dryopteridaceae	N	10	0	10
Pontederia cordata	L.	Pontederiaceae	A	2	0	2
Populus nigra aggr.		Salicaceae	N	2	0	2
Portulaca oleracea	L.	Portulacaceae	N	0	32	32
Potamogeton natans	L.	Potamogetonaceae	N	1	0	1
Potentilla anserina	L.	Rosaceae	N	0	3	3
Potentilla argentea	L.	Rosaceae	N	2	0	2
Potentilla fruticosa	(L.) Rydb.	Rosaceae	A	5	0	5
Potentilla recta	L.	Rosaceae	N	1	0	1
Potentilla reptans	L.	Rosaceae	N	0	34	34
Potentilla sterilis	(L.) Garcke	Rosaceae	N	0	18	18
Potentilla verna	L.	Rosaceae	N	2	0	2
Primula acaulis	Huds.	Primulaceae	N	85	0	85
Primula elatior	(L.) L.	Primulaceae	N	10	1	11
Primula veris	L.	Primulaceae	N	3	0	3
Prunella grandiflora	(L.) Scholler	Lamiaceae	N	2	0	2
Prunella vulgaris	L.	Lamiaceae	N	8	36	44
Prunus armeniaca	L.	Rosaceae	A	16	0	16
Prunus avium	L.	Rosaceae	N	16	1	17
Prunus cerasifera	Ehrh.	Rosaceae	A	4	0	4
Prunus cerasus	L.	Rosaceae	A	1	0	1
Prunus domestica	L.	Rosaceae	A	19	0	19
Prunus dulcis	(Mill.) D. A. Webb	Rosaceae	A	1	0	1
Prunus kurilensis	(Miyabe) Miyabe	Rosaceae	A	1	0	1
Prunus laurocerasus	L.	Rosaceae	A	17	0	17
Prunus lusitanica	L.	Rosaceae	A	2	0	2
Prunus padus	L.	Rosaceae	N	3	0	3
Prunus persica	(L.) Batsch	Rosaceae	A	6	0	6
Prunus serrulata	Lindl.	Rosaceae	A	3	0	3
Prunus spinosa	L.	Rosaceae	N	5	0	5
Prunus triloba	Lindl.	Rosaceae	A	1	0	1
Pseudolysimachion spicatum	(L.) Opiz	Plantaginaceae	N	3	0	3
Pseudosasa japonica	(Steud.) Nakai	Poaceae	A	8	0	8
Pulmonaria obscura	Dumort.	Boraginaceae	N	2	0	2
Pulmonaria officinalis	L.	Boraginaceae	N	2	0	2
Pulsatilla vulgaris	L.	Ranunculaceae	N	6	0	6
Punica granatum	L.	Lythraceae	A	1	0	1
Pyracantha coccinea	M. Roem.	Rosaceae	A	6	0	6
Pyrus communis	L.	Rosaceae	A	15	0	15
Quercus robur	L.	Fagaceae	N	2	5	7
Ranunculus acris subsp. friesianus	(Jord.) Syme	Ranunculaceae	N	5	13	18
Ranunculus auricomus aggr.		Ranunculaceae	N	0	1	1
Ranunculus bulbosus	L.	Ranunculaceae	N	1	0	1
Ranunculus ficaria	L.	Ranunculaceae	N	0	69	69
Ranunculus gramineus	L.	Ranunculaceae	N	1	0	1
Ranunculus lingua	L.	Ranunculaceae	N	2	0	2
Ranunculus repens	L.	Ranunculaceae	N	0	53	53
Raphanus sativus	L.	Brassicaceae	A	27	0	27
Reseda lutea	L.	Resedaceae	N	3	0	3
Rheum rhabarbarum	L.	Polygonaceae	A	29	0	29
Rhodiola rosea	L.	Crassulaceae	N	1	0	1
Rhododendron Catawbiense Group		Ericaceae	A	19	0	19
Rhododendron luteum	Sweet	Ericaceae	A	3	0	3
Rhus typhina	L.	Anacardiaceae	A	1	0	1
Ribes × nidigrolaria	Rud. Bauer & A. Bauer	Grossulariaceae	A	8	0	8
Ribes alpinum	L.	Grossulariaceae	N	2	0	2
Ribes aureum	Pursh	Grossulariaceae	A	2	0	2
Ribes nigrum	L.	Grossulariaceae	A	15	0	15
Ribes rubrum	L.	Grossulariaceae	A	56	0	56
Ribes sanguineum	Pursh	Grossulariaceae	A	1	0	1
Ribes uva-crispa	L.	Grossulariaceae	N	24	0	24
Ricinus communis	L.	Euphorbiaceae	A	3	0	3
Robinia pseudoacacia	L.	Fabaceae	A	2	3	5
Rodgersia aesculifolia	Batalin	Saxifragaceae	A	1	0	1
Rodgersia podophylla	A. Gray	Saxifragaceae	A	1	0	1
Rorippa sylvestris	(L.) Besser	Brassicaceae	N	0	2	2
Rosa arvensis	Huds.	Rosaceae	N	0	1	1
Rosa canina aggr.		Rosaceae	N	11	10	21
Rosa CV		Rosaceae	A	68	0	68
Rosa glauca	Pourr.	Rosaceae	N	5	0	5
Rosa multiflora	Thunb.	Rosaceae	A	2	0	2
Rosa pendulina	L.	Rosaceae	N	1	0	1
Rosa rubiginosa aggr.		Rosaceae	N	3	0	3
Rosa rugosa	Thunb.	Rosaceae	A	1	0	1
Rosa spinosissima	L.	Rosaceae	N	4	0	4
Rosmarinus officinalis	L.	Lamiaceae	A	43	0	43
Rubus armeniacus	Focke	Rosaceae	A	47	1	48
Rubus caesius	L.	Rosaceae	N	0	11	11
Rubus idaeus	L.	Rosaceae	N	47	0	47
Rubus phoenicolasius	Maxim.	Rosaceae	A	1	0	1
Rudbeckia hirta	L.	Asteraceae	A	20	0	20
Rudbeckia laciniata	L.	Asteraceae	A	3	0	3
Rumex acetosa	L.	Polygonaceae	N	0	4	4
Rumex obtusifolius	L.	Polygonaceae	N	0	18	18
Rumex sanguineus	L.	Polygonaceae	N	10	0	10
Ruscus aculeatus	L.	Asparagaceae	N	1	0	1
Ruta graveolens	L.	Rutaceae	N	9	0	9
Sagina procumbens	L.	Caryophyllaceae	N	0	20	20
Sagittaria latifolia	Willd.	Alismataceae	A	1	0	1
Salix × fragilis	L.	Salicaceae	A	1	0	1
Salix caprea	L.	Salicaceae	N	4	13	17
Salix gracilistyla	Miq.	Salicaceae	A	1	0	1
Salix integra	Thunb.	Salicaceae	A	1	0	1
Salix matsudana	L.	Salicaceae	A	1	0	1
Salix purpurea	L.	Salicaceae	N	1	0	1
Salvia × jamensis	J. Compton	Lamiaceae	A	1	0	1
Salvia × sylvestris	L.	Lamiaceae	A	3	0	3
Salvia africana	L.	Lamiaceae	A	1	0	1
Salvia apiana	Jeps.	Lamiaceae	A	1	0	1
Salvia elegans	Vahl	Lamiaceae	A	9	0	9
Salvia farinacea	Benth.	Lamiaceae	A	1	0	1
Salvia glutinosa	L.	Lamiaceae	N	1	0	1
Salvia officinalis	L.	Lamiaceae	A	33	0	33
Salvia patens	Cav.	Lamiaceae	A	5	0	5
Salvia pratensis	L.	Lamiaceae	N	2	0	2
Salvia sclarea	L.	Lamiaceae	A	4	0	4
Salvia verticillata	L.	Lamiaceae	A	1	0	1
Sambucus nigra	L.	Adoxaceae	N	27	11	38
Sanguisorba minor	Scop.	Rosaceae	N	8	0	8
Sanguisorba officinalis	L.	Rosaceae	N	3	0	3
Santolina chamaecyparissus	L.	Asteraceae	A	3	0	3
Santolina rosmarinifolia	L.	Asteraceae	A	2	0	2
Sanvitalia procumbens	Lam.	Asteraceae	A	4	0	4
Saponaria ocymoides	L.	Caryophyllaceae	N	4	0	4
Saponaria officinalis	L.	Caryophyllaceae	N	5	10	15
Sarracenia purpurea	L.	Sarraceniaceae	A	1	0	1
Satureja hortensis	L.	Lamiaceae	A	7	0	7
Satureja montana	L.	Lamiaceae	A	9	0	9
Saxifraga × arendsii	Engl.	Saxifragaceae	A	10	0	10
Saxifraga paniculata	Mill.	Saxifragaceae	N	5	0	5
Saxifraga stolonifera	Meerb.	Saxifragaceae	A	1	0	1
Saxifraga tridactylites	L.	Saxifragaceae	N	0	4	4
Saxifraga umbrosa	L.	Saxifragaceae	A	4	0	4
Scabiosa caucasica	(M. Bieb.) Greuter & Burdet	Caprifoliaceae	A	4	0	4
Scabiosa columbaria	L.	Caprifoliaceae	N	4	0	4
Scabiosa ochroleuca	L.	Caprifoliaceae	A	1	0	1
Schoenoplectus lacustris	(L.) Palla	Cyperaceae	N	1	0	1
Schoenoplectus mucronatus	(L.) Palla	Cyperaceae	N	0	1	1
Scilla forbesii	(Baker) Speta	Asparagaceae	A	29	0	29
Scilla siberica	Haw.	Asparagaceae	A	21	0	21
Scorzonera hispanica	L.	Asteraceae	A	4	0	4
Scrophularia nodosa	L.	Scrophulariaceae	N	0	4	4
Scutellaria altissima	L.	Lamiaceae	A	1	0	1
Sechium edule	(Jacq.) Sw.	Cucurbitaceae	A	3	0	3
Securigera varia	(L.) Lassen	Fabaceae	N	0	1	1
Sedum acre	L.	Crassulaceae	N	0	1	1
Sedum album	L.	Crassulaceae	N	9	1	10
Sedum floriferum	Fisch.	Crassulaceae	A	14	0	14
Sedum hispanicum	L.	Crassulaceae	N	0	2	2
Sedum rupestre aggr.		Crassulaceae	N	14	0	14
Sedum sexangulare	L.	Crassulaceae	N	1	18	19
Sedum sieboldii	Regel	Crassulaceae	A	2	0	2
Sedum spurium	M. Bieb.	Crassulaceae	A	14	0	14
Sedum telephium	L.	Crassulaceae	N	36	0	36
Sempervivum arachnoideum	L.	Crassulaceae	N	1	0	1
Sempervivum montanum	L.	Crassulaceae	N	1	0	1
Sempervivum tectorum	L.	Crassulaceae	N	18	0	18
Senecio cineraria	DC.	Asteraceae	A	3	0	3
Senecio jacobaea	L.	Asteraceae	N	1	3	4
Senecio vulgaris	L.	Asteraceae	N	0	32	32
Setaria italica	(L.) P. Beauv.	Poaceae	A	0	1	1
Setaria pumila	(Poir.) Roem. & Schult.	Poaceae	N	0	2	2
Setaria verticillata	(L.) P. Beauv.	Poaceae	N	0	2	2
Setaria viridis	(L.) P. Beauv.	Poaceae	N	0	7	7
Sherardia arvensis	L.	Rubiaceae	N	0	2	2
Silene armeria	L.	Caryophyllaceae	N	1	0	1
Silene chalcedonica	(L.) E. H. L. Krause	Caryophyllaceae	A	3	0	3
Silene coronaria	(L.) Clairv.	Caryophyllaceae	N	11	0	11
Silene dioica	(L.) Clairv.	Caryophyllaceae	N	3	4	7
Silene flos-cuculi	(L.) Clairv.	Caryophyllaceae	N	3	0	3
Silene nutans	L.	Caryophyllaceae	N	0	1	1
Silene pratensis	(Rafn) Godr.	Caryophyllaceae	N	0	1	1
Silene vulgaris	(Moench) Garcke	Caryophyllaceae	N	3	0	3
Silybum marianum	(L.) Gaertn.	Asteraceae	A	1	0	1
Sinapis alba	L.	Brassicaceae	N	8	1	9
Sium sisarum	L.	Apiaceae	A	1	0	1
Skimmia japonica	Thunb.	Rutaceae	A	3	0	3
Solanum dulcamara	L.	Solanaceae	N	0	5	5
Solanum jasminoides	J. Paxton	Solanaceae	A	3	0	3
Solanum melongena	L.	Solanaceae	A	11	0	11
Solanum muricatum	Aiton	Solanaceae	A	1	0	1
Solanum nigrum	L.	Solanaceae	N	0	8	8
Solanum sisymbriifolium	Lam.	Solanaceae	A	1	0	1
Solanum tuberosum	L.	Solanaceae	A	34	0	34
Solenostemon scutellarioides	(L.) R. Br.	Lamiaceae	A	1	0	1
Solidago canadensis	L.	Asteraceae	A	15	0	15
Solidago gigantea	Aiton	Asteraceae	A	7	0	7
Solidago virgaurea	L.	Asteraceae	N	2	0	2
Sonchus arvensis	L.	Asteraceae	N	0	14	14
Sonchus asper	Hill	Asteraceae	N	0	56	56
Sonchus oleraceus	L.	Asteraceae	N	0	28	28
Sorbus aria	(L.) Crantz	Rosaceae	N	1	0	1
Sorbus aucuparia	L.	Rosaceae	N	5	0	5
Sorbus intermedia	(Ehrh.) Pers.	Rosaceae	A	2	0	2
Sorghum bicolor	(L.) Moench	Poaceae	A	1	0	1
Sorghum halepense	(L.) Pers.	Poaceae	A	0	1	1
Spinacia oleracea	L.	Amaranthaceae	A	1	0	1
Spiraea × arguta	Zabel	Rosaceae	A	1	0	1
Spiraea × cinerea	Zabel	Rosaceae	A	3	0	3
Spiraea × vanhouttei	(Briot) Zabel	Rosaceae	A	10	0	10
Spiraea betulifolia	Pall.	Rosaceae	A	4	0	4
Spiraea japonica	L. f.	Rosaceae	A	5	0	5
Spirodela polyrhiza	(L.) Schleid.	Araceae	N	2	0	2
Stachys affinis	Bunge	Lamiaceae	A	2	0	2
Stachys byzantina	K. Koch	Lamiaceae	A	4	0	4
Stachys palustris	L.	Lamiaceae	N	0	1	1
Stachys recta	L.	Lamiaceae	N	2	0	2
Stachys sylvatica	L.	Lamiaceae	N	0	6	6
Staphylea pinnata	L.	Staphyleaceae	N	1	0	1
Stellaria media	(L.) Vill.	Caryophyllaceae	N	0	25	25
Stevia rebaudiana	(Bertoni) Bertoni	Asteraceae	A	1	0	1
Symphoricarpos × chenaultii	Rehder	Caprifoliaceae	A	4	0	4
Symphoricarpos albus	(L.) S. F. Blake	Caprifoliaceae	A	9	0	9
Symphytum asperum	Lepech.	Boraginaceae	A	2	0	2
Symphytum grandiflorum	DC.	Boraginaceae	A	5	0	5
Symphytum officinale	L.	Boraginaceae	N	16	0	16
Syringa meyeri	Turcz.	Oleaceae	A	4	0	4
Syringa vulgaris	L.	Oleaceae	A	30	0	30
Tagetes erecta	L.	Asteraceae	A	3	0	3
Tagetes patula	L.	Asteraceae	A	21	0	21
Tagetes tenuifolia	Cav.	Asteraceae	A	2	0	2
Tamarix parviflora	DC.	Tamaricaceae	A	3	0	3
Tanacetum corymbosum	(L.) Sch. Bip.	Asteraceae	N	1	0	1
Tanacetum parthenium	(L.) Sch. Bip.	Asteraceae	A	19	0	19
Tanacetum vulgare	L.	Asteraceae	N	18	0	18
Taraxacum officinale aggr.		Asteraceae	N	0	85	85
Taxus baccata	L.	Taxaceae	N	16	0	16
Telekia speciosa	(Schreb.) Baumg.	Asteraceae	A	2	0	2
Tellima grandiflora	(Pursh) Lindl.	Saxifragaceae	A	1	0	1
Tetragonia tetragonoides	(Pall.) Kuntze	Aizoaceae	A	1	0	1
Teucrium chamaedrys	L.	Lamiaceae	N	4	0	4
Thalictrum aquilegiifolium	L.	Ranunculaceae	N	5	0	5
Thalictrum minus	L.	Ranunculaceae	N	1	0	1
Thelypteris palustris	Schott	Thelypteridaceae	N	1	0	1
Thuja occidentalis	L.	Cupressaceae	A	20	0	20
Thunbergia alata	Bojer ex Sims	Acanthaceae	A	3	0	3
Thymus × citriodorus	(Pers.) Schreb.	Lamiaceae	A	3	0	3
Thymus longicaulis	C. Presl	Lamiaceae	N	9	0	9
Thymus pulegioides	L.	Lamiaceae	N	13	0	13
Thymus vulgaris	L.	Lamiaceae	A	22	0	22
Tiarella cordifolia	L.	Saxifragaceae	A	1	0	1
Tilia cordata	Mill.	Malvaceae	N	1	0	1
Tilia platyphyllos	Scop.	Malvaceae	N	2	2	4
Tithonia rotundifolia	(Mill.) S. F. Blake	Asteraceae	A	1	0	1
Tofieldia calyculata	(L.) Wahlenb.	Tofieldiaceae	N	1	0	1
Torenia fournieri	Linden ex E. Fourn.	Linderniaceae	A	1	0	1
Torilis japonica	(Houtt.) DC.	Apiaceae	N	0	1	1
Trachycarpus fortunei	(Hook.) H. Wendl.	Arecaceae	A	2	2	4
Trachymene coerulea	Graham	Apiaceae	A	1	0	1
Tradescantia pallida	(Rose) D. R. Hunt	Commelinaceae	A	1	0	1
Tradescantia virginiana	L.	Commelinaceae	A	12	0	12
Tragopogon porrifolius	L.	Asteraceae	A	3	0	3
Tragopogon pratensis	L.	Asteraceae	N	4	3	7
Tricyrtis hirta	(Thunb.) Hook.	Liliaceae	A	2	0	2
Trifolium campestre	Schreb.	Fabaceae	A	0	1	1
Trifolium dubium	Sibth.	Fabaceae	N	0	8	8
Trifolium incarnatum	L.	Fabaceae	A	1	0	1
Trifolium pratense	L.	Fabaceae	N	12	6	18
Trifolium repens	L.	Fabaceae	N	0	66	66
Tripleurospermum inodorum	(L.) Sch. Bip.	Asteraceae	N	0	1	1
Trisetum flavescens	(L.) P. Beauv.	Poaceae	N	3	2	5
Triticum aestivum	L.	Poaceae	A	0	3	3
Trollius europaeus	L.	Ranunculaceae	N	2	0	2
Tropaeolum majus	L.	Tropaeolaceae	A	31	0	31
Tropaeolum tuberosum	Ruiz & Pav.	Tropaeolaceae	A	1	0	1
Tulipa CV		Liliaceae	A	48	0	48
Tulipa turkestanica	(Regel) Regel	Liliaceae	A	3	0	3
Tussilago farfara	L.	Asteraceae	N	0	5	5
Typha latifolia	L.	Typhaceae	N	2	0	2
Typha minima	Hoppe	Typhaceae	N	1	0	1
Ulmus glabra	Huds.	Ulmaceae	N	0	2	2
Ulmus minor	Mill.	Ulmaceae	N	0	1	1
Urtica dioica	L.	Urticaceae	N	0	29	29
Utricularia vulgaris aggr.		Lentibulariaceae	N	1	0	1
Vaccaria hispanica	(Mill.) Rauschert	Caryophyllaceae	N	3	0	3
Vaccinium corymbosum	L.	Ericaceae	A	23	0	23
Vaccinium vitis-idaea	L.	Ericaceae	N	4	0	4
Valeriana officinalis	L.	Caprifoliaceae	N	8	4	12
Valerianella locusta	(L.) Laterr.	Caprifoliaceae	N	32	0	32
Verbascum blattaria	L.	Scrophulariaceae	N	1	0	1
Verbascum lychnitis	L.	Scrophulariaceae	N	1	0	1
Verbascum nigrum	L.	Scrophulariaceae	N	6	1	7
Verbascum phoeniceum	L.	Scrophulariaceae	A	1	0	1
Verbascum thapsus	L.	Scrophulariaceae	N	0	24	24
Verbena × hybrida	(Groenl. & Rümpler) G. L. Nesom & Pruski	Verbenaceae	A	3	0	3
Verbena bonariensis	L.	Verbenaceae	A	5	0	5
Verbena officinalis	L.	Verbenaceae	N	0	10	10
Verbena rigida	Spreng.	Verbenaceae	A	2	0	2
Veronica agrestis	L.	Plantaginaceae	N	0	9	9
Veronica arvensis	L.	Plantaginaceae	N	0	26	26
Veronica beccabunga	L.	Plantaginaceae	N	3	0	3
Veronica chamaedrys	L.	Plantaginaceae	N	9	30	39
Veronica filiformis	Sm.	Plantaginaceae	A	0	31	31
Veronica hederifolia	L.	Plantaginaceae	N	0	59	59
Veronica montana	L.	Plantaginaceae	N	0	2	2
Veronica peregrina	L.	Plantaginaceae	A	0	25	25
Veronica persica	Poir.	Plantaginaceae	A	0	52	52
Veronica polita	Fr.	Plantaginaceae	N	0	2	2
Veronica serpyllifolia	L.	Plantaginaceae	N	4	43	47
Viburnum × bodnantense	Stearn	Adoxaceae	A	5	0	5
Viburnum carlesii	Hemsl.	Adoxaceae	A	2	0	2
Viburnum lantana	L.	Adoxaceae	N	6	0	6
Viburnum opulus	L.	Adoxaceae	N	7	0	7
Viburnum plicatum	Thunb.	Adoxaceae	A	1	0	1
Viburnum rhytidophyllum	Hemsl.	Adoxaceae	A	1	0	1
Viburnum tinus	L.	Adoxaceae	A	1	0	1
Vicia cracca	L.	Fabaceae	N	0	2	2
Vicia faba	L.	Fabaceae	A	2	0	2
Vicia sativa	L.	Fabaceae	N	0	1	1
Vicia sepium	L.	Fabaceae	N	0	50	50
Vinca major	L.	Apocynaceae	A	5	0	5
Vinca minor	L.	Apocynaceae	N	16	0	16
Viola × wittrockiana	Gams	Violaceae	A	8	0	8
Viola alba	Besser	Violaceae	N	1	3	4
Viola cornuta	L.	Violaceae	A	18	0	18
Viola elatior	Fr.	Violaceae	N	1	0	1
Viola hirta	L.	Violaceae	N	0	4	4
Viola odorata	L.	Violaceae	N	0	33	33
Viola reichenbachiana	Boreau	Violaceae	N	0	41	41
Viola riviniana	Rchb.	Violaceae	N	0	3	3
Viola tricolor aggr.		Violaceae	N	18	0	18
Vitex agnus-castus	L.	Lamiaceae	A	2	0	2
Vitis vinifera	L.	Vitaceae	A	47	0	47
Waldsteinia ternata	(Stephan) Fritsch	Rosaceae	A	2	0	2
Weigela florida	(Bunge) A. DC.	Caprifoliaceae	A	12	0	12
Wisteria sinensis	(Sims) Sweet	Fabaceae	A	16	0	16
Yucca filamentosa	L.	Asparagaceae	A	10	0	10
Yucca gloriosa	L.	Asparagaceae	A	2	0	2
Zantedeschia albomaculata	(Hook.) Baill.	Araceae	A	3	0	3
Zanthoxylum piperitum	Maxim.	Rutaceae	A	2	0	2
Zea mays	L.	Poaceae	A	17	0	17
Zelkova serrata	(Thunb.) Makino	Ulmaceae	A	1	0	1
Zinnia elegans	L.	Asteraceae	A	19	0	19
Total				6620	3481	10101
