# Supplementary material for: A comprehensive dataset on cultivated and spontaneously growing vascular plants in urban gardens
Source: Data Brief. 2019 May 23;25:103982. doi: 10.1016/j.dib.2019.103982 (PMC6545399; doi:10.1016/j.dib.2019.103982)
Supplement: Supplementary file 1 — Multimedia component 1 [file mmc1.pdf]

Conflict of Interest and Authorship Conformation Form

Please check the following as appropriate:

- o All authors have participated in (a) conception and design, or analysis and interpretation of the data; (b) drafting the article or revising it critically for important intellectual content; and (c) approval of the final version.

- o This manuscript has not been submitted to, nor is under review at, another journal or other publishing venue.

- o The authors have no affiliation with any organization with a direct or indirect financial interest in the subject matter discussed in the manuscript

- o The following authors have affiliations with organizations with direct or indirect financial interest in the subject matter discussed in the manuscript:

Author's name	Affiliation
David Frey	Swiss Federal Research Institute WSL & ETH Zurich
Marco Moretti	Swiss Federal Research Institute WSL
